# Supplementary material for: Lin28B-high breast cancer cells promote immune suppression in the lung pre-metastatic niche via exosomes and support cancer progression
Source: Nat Commun. 2022 Feb 16;13:897. doi: 10.1038/s41467-022-28438-x (PMC8850492; doi:10.1038/s41467-022-28438-x)
Supplement: Supplementary file 1 — Supplementary Information [file 41467_2022_28438_MOESM1_ESM.pdf]

Supplementary Information

Supplementary Information contains 12 Supplementary Figures and 8 Supplementary Tables.

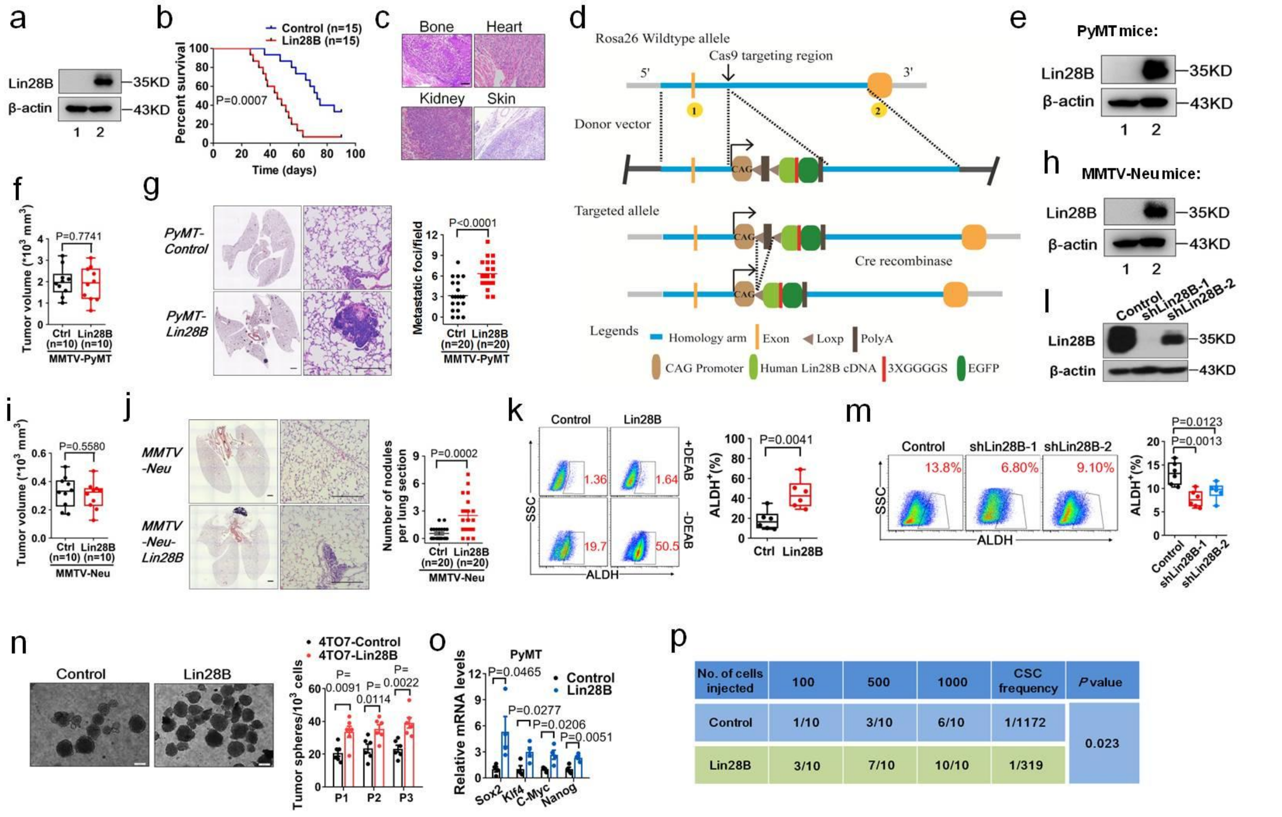

Supplementary Figure 1. Lin28B promotes lung metastasis and stem cell properties of breast tumor cells. (a) Lin28B expression was analyzed in 4TO7-Control (line 1) and 4TO7-Lin28B (line 2) cells. (b) Kaplan-Meier survival plots of the tumor-bearing mice (*n*=15). (c) H&E staining sections of metastasis for 4TO7-Lin28B mice. Scale bars: 200 µm. (d) Schematic generation of Lin28B knock-in mice (*Lin28B^{KI}*) through CRISPR/Cas9-mediated genome editing. (e) Lin28B expression was analyzed in *PyMT-Control* (line 1) and *PyMT-Lin28B* (line 2) tumors. (f) Tumor volume of 11-week-old indicated mice (*n*=10). (g) Histologic image and quantitation of lung metastasis of 13-week-old mice (*n*=20). Scale bars: 200 µm. (h) Lin28B expression was analyzed in *MMTV-Neu-Control* (line 1) and *MMTV-Neu-Lin28B* (line 2) tumors. (i) Tumor volume of 5-month-old indicated mice (*n*=10). (j) Image and quantitation of lung metastasis of 6-month-old mice (*n*=20). Scale bars: 200 µm. (k) Flow cytometry analyses of ALDH^{+} CSCs in 4TO7 cells (*n*=6 culturing experiments). (l) The knockdown effect of Lin28B was detected in MDA-MB-231 cells. (m) Flow cytometry analyses of ALDH^{+} CSCs in MDA-MB-231 cells transfected as

indicated (*n*=6 culturing experiments). (n) Images and quantitation of tumorspheres in 4TO7-derivative cells (*n*=6 culturing experiments). Scale bar: 100 µm. (o) Stem gene were detected by qRT-PCR in PyMT tumors (*n*=4 mice in each group). (p) In vivo tumor formation in mice inoculated with serial dilutions of 4TO7-derivative cells (*n*=10).

For panel (f), (i), (k) and (m), boxes represent data within the 25th to 75th percentiles. Whiskers depict the range of all data points. Horizontal lines within boxes represent mean values. Experiments in (a), (c), (e), (h) and (l) were repeated three times independently with similar results; data from one representative experiment are shown. In (b, f, g, i, j, k, m, n, o, p), data are presented as mean ± SEM. A two-sided log-rank test was used for statistical analysis of (b), chi-squared test for (p), one-way ANOVA followed by multiple comparisons for (g) and (j), two-tailed Student's t-test for (f), (i), (k), (m), (n) and (o) *p < 0.05, **p < 0.01, ***p < 0.001. Source data are provided as a Source Data file.

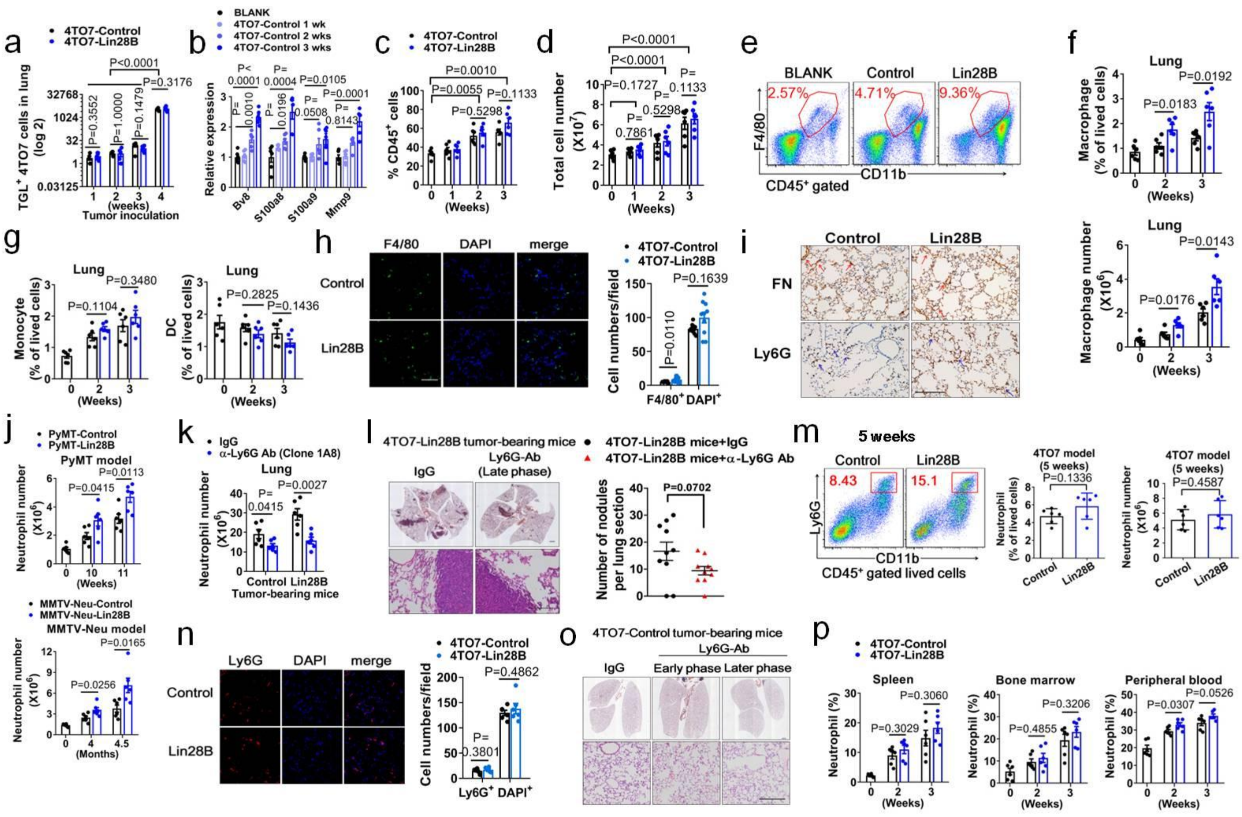

Supplementary Figure 2. Lin28B expression promotes neutrophil accumulation in the pre-metastatic niche, but not other cells.

(a) qRT-PCR was used to detect the number of tumor cells in the pre-metastatic lung (*n*=6 mice in each group). (b) A similar experiment as in Fig. 2a, except that the representative genes were detected at different time points (*n*=6 mice in each group). (c and d) The $CD45^{+}$ cell proportion (c) and the numbers (d) in the indicated mice lungs (*n*=6 mice in each group). (e) Representative flow cytometry plots of $CD11b^{+}F4/80^{+}$ macrophages. (f-g) Proportions or numbers of macrophages, monocytes and DCs in the pre-metastatic lung (*n*=6 mice in each group). DCs: dendritic cells. (h) IF analysis and quantitation of F4/80 expression in the pre-metastatic lung (*n*=10 random microscopic fields (RMFs) from 3 mice each group). Scale bar: 100 μm. (i) IHC staining of FN and Ly6G in the pre-metastatic lung. Scale bars: 200 μm. (j) Lung neutrophils were detected in the pre-metastatic lung in *PyMT* and *MMTV-Neu* mice (*n*=6 mice in each group). (k) Lung neutrophils were detected in mice treated with neutrophil-clearance antibody (*n*=6 mice in each group). (l) H&E and quantification of lung metastasis in 4TO7-Lin28B mice in which the neutrophils were depleted in late phase (*n* = 10 RMFs from 3 mice). Scale bars: 200 μm. (m) The lung neutrophils

were analyzed in the tumor-bearing mice (*n*=6 mice in each group). (n) IF analysis and quantitation of Ly6G expression in the lung at 5 weeks after tumor inoculation (*n*=6 RMFs from 3 mice). Scale bar: 100 μm. (o) H&E staining in lung tissues in 4TO7-Control mice with neutrophil depletion. Scale bars: 200 μm. (p) Neutrophils were detected in the tissues in the indicated mice (*n* = 6 mice in each group).

Data are presented as the mean ± SEM, and p-values were calculated using two-tailed Student's *t*-test. *p < 0.05, **p < 0.01, ***p < 0.001, ns, no significance. For panel i and o, IHC or H&E staining was performed on lung sections of three mice in each group with similar results. Source data are provided as a Source Data file.

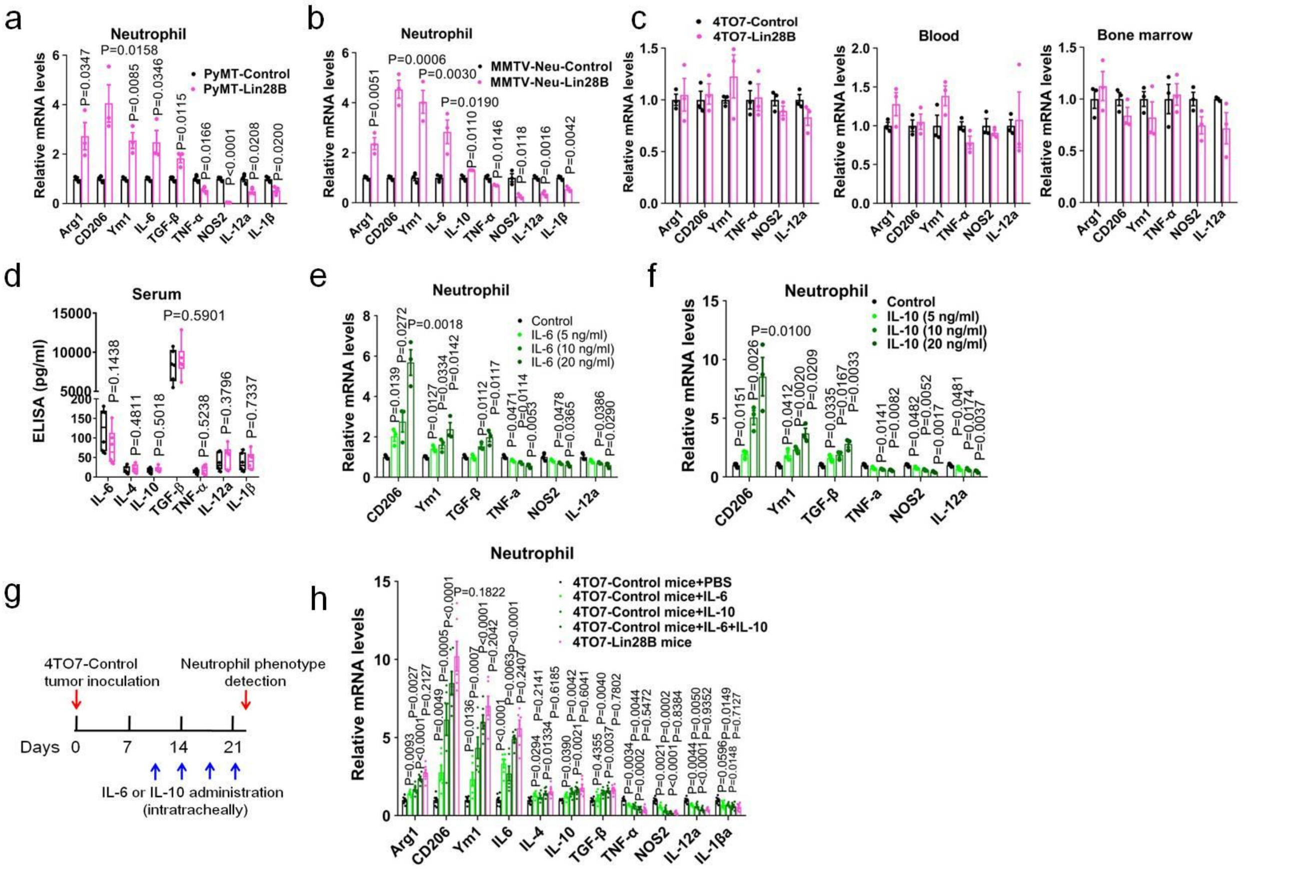

Supplementary Figure 3. IL-6 and IL-10 were identified as promoters of neutrophil N2 conversion in the pre-metastatic niche.

(a) Quantification of N1/N2 marker genes in neutrophils collected from the pre-metastatic lung in 10-week old *PyMT-Lin28B* and the control mice. GAPDH served as internal control (*n*=3 mice in each group). (b) Quantification of N1/N2 marker genes in neutrophils collected from the pre-metastatic lung in 4-month old *MMTV-Neu-Lin28B* and the control mice. GAPDH served as internal control (*n*=3 mice in each group). (c) Quantification of N1/N2 marker genes of neutrophils purified from blood, spleen, or bone marrow of tumor-bearing mice at 3 weeks after tumor inoculation (*n*=3 mice in each group). (d) Quantification of indicated cytokines in the serum of tumor-bearing mice at 3 weeks after tumor inoculation. Boxes represent data within the 25th to 75th percentiles. Whiskers depict the range of all data points. Horizontal lines within boxes represent mean values (*n* = 6 mice in each group). (e) Quantification of N1/N2 marker genes of in vitro-cultured bone marrow neutrophils stimulated with different dosages of IL-6 as indicated. GAPDH served as internal control (*n*=3 culturing experiments). (f) A similar experiment as in

panel e, except that IL-6 was replaced with IL-10. (g) Schematic diagram for inducing N2 conversion of lung neutrophils in 4TO7-Control tumor-bearing mice. (h) Neutrophil phenotypes were detected in the lungs of tumor-bearing mice treated as indicated in panel g. GAPDH served as internal control (*n*=6 mice in each group).

Data are presented as the mean ± SEM, and p-values were calculated using two-tailed Student's *t*-test. *p < 0.05, **p < 0.01, ***p < 0.001, ns, no significance. Source data are provided as a Source Data file.

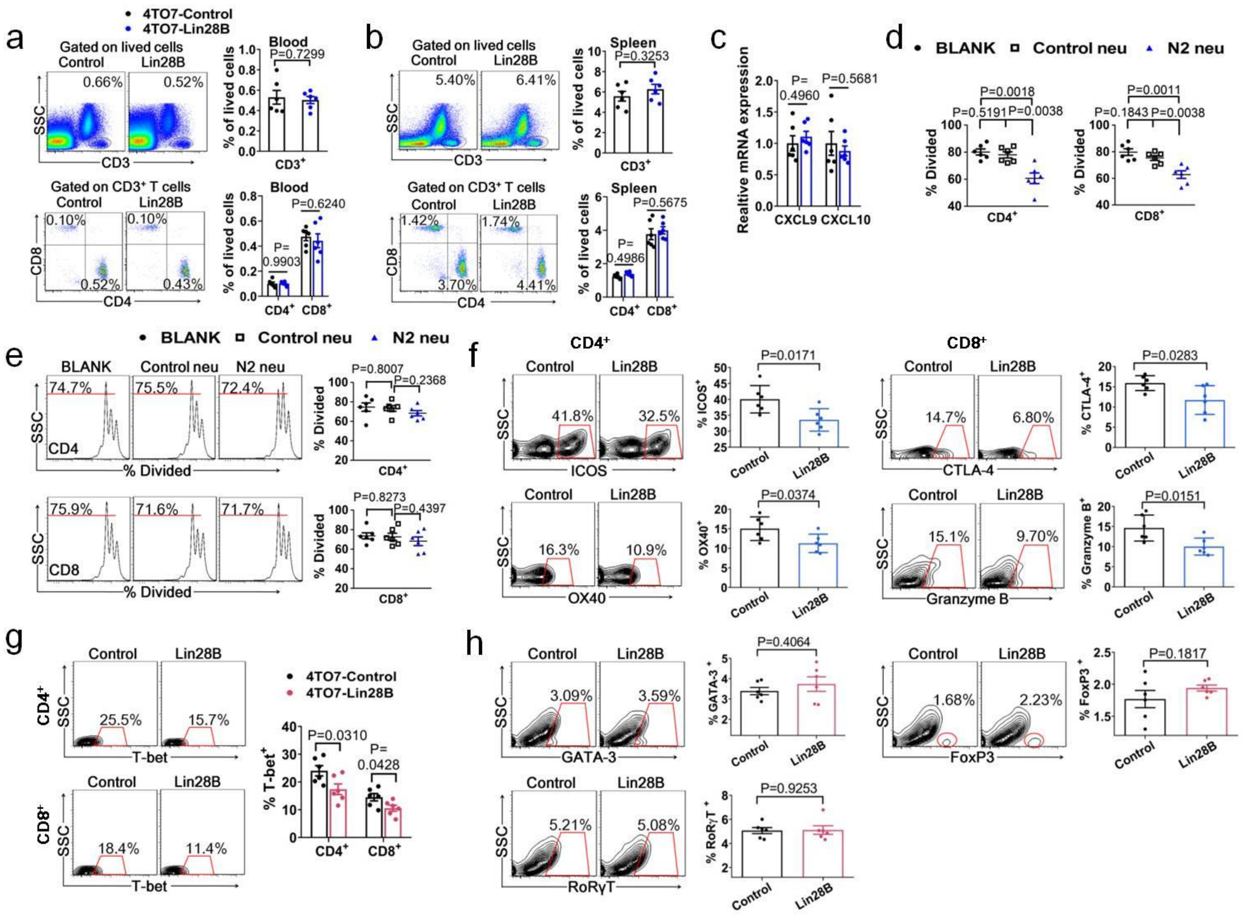

Supplementary Figure 4. N2-converted neutrophils suppress CD4+ and CD8+ T cell activity. (a and b) Representative flow cytometry plots and percentage of the indicated T cells in the blood (a) and spleen (b) of tumor-bearing mice (*n* = 6 mice in each group). (c) Quantification of CXCL9 and CXCL10 mRNA in the pre-metastatic lung of tumor-bearing mice (*n*=6 mice in each group). (d) CFSE-labeled naïve T cells (CD4+ and CD8+) isolated from bone marrow of BALB/c mice were stimulated with anti-CD3/CD28 Abs and mixed without or with neutrophils purified from lung of tumor-bearing mice by FACS at a 1:1 ratio. The percentage of proliferating T cells was shown (*n* = 6 culturing experiments). (e) Flow cytometric analysis of T cell proliferation in the presence of N2 neutrophils using a transwell system (*n*=6 culturing experiments). (f) The expression of ICOS and OX40 in CD4+ T cells, and the expression of CTLA-4 and Granzyme B in CD8+ T cells were analyzed in the pre-metastatic lung (*n*=6 mice in each group). (g) The expression of T-bet was analyzed in CD4+ and CD8+ T cells isolated from lungs of tumor-bearing mice by FACS. Representative contour plots and quantitative data are shown (*n*=6 mice in each

group). (h) The expression of GATA-3, FoxP3, and RoRγT were analyzed in $CD4^+$ T cells isolated from the pre-metastatic lungs of tumor-bearing mice (*n*=6 mice in each group). The lung cells (neutrophils, $CD4^+$ and $CD8^+$ T cells) were isolated from tumor-bearing mice at 3weeks after tumor inoculation. Data are presented as the mean ± SEM, and p-values were calculated using two-tailed Student's *t*-test. *$p < 0.05$, **$p < 0.01$, ns, no significance. Source data are provided as a Source Data file.

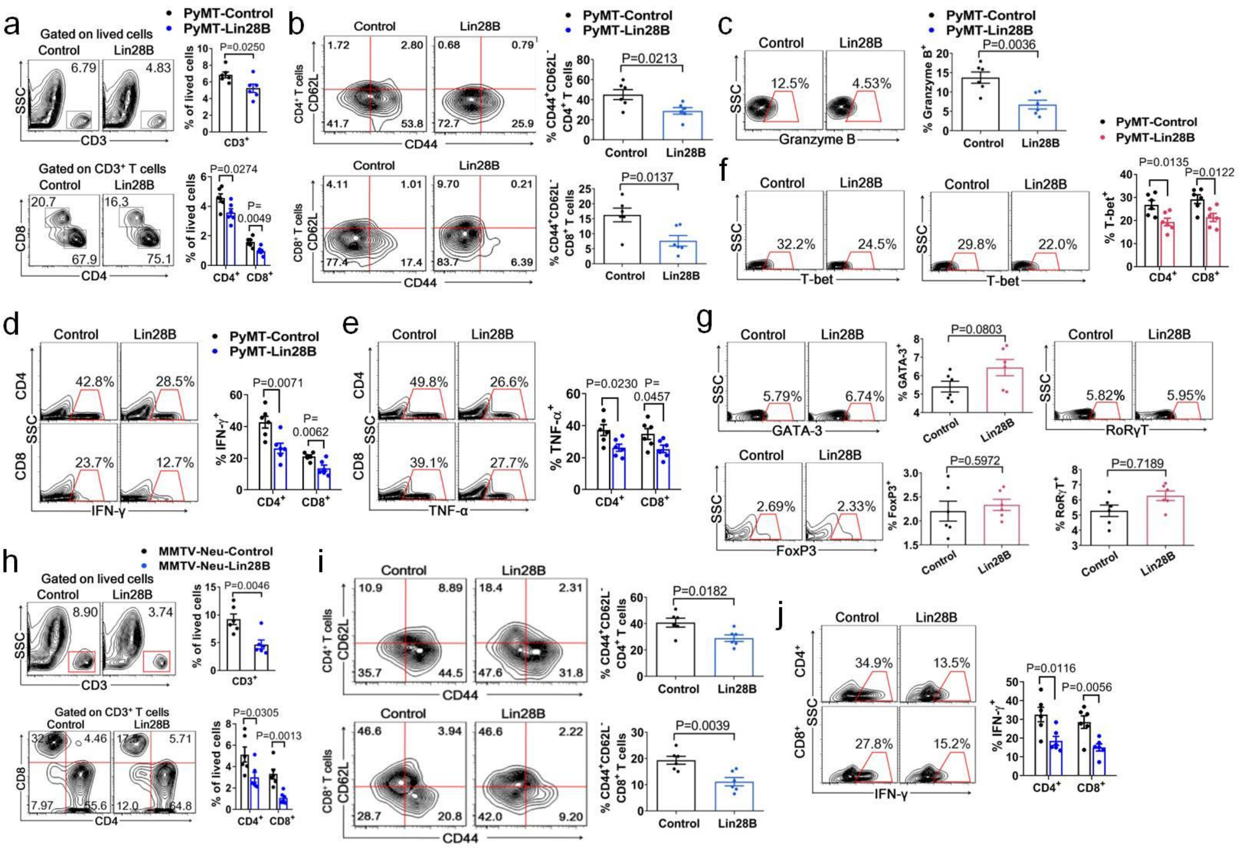

Supplementary Figure 5. The immune-suppressive activity of Lin28B was confirmed in *PyMT* model.

(a) Representative flow cytometry plots and percentage of the indicated T cell subsets in the lungs of 11-week old *PyMT-Control* and *PyMT-Lin28B* mice (*n*=6 mice in each group). (b) The CD4$^+$ and CD8$^+$ T cells prepared as in panel a were further analyzed for CD44 and CD62L expression (*n*=6 mice in each group). (c) CD8$^+$ T cells prepared as in panel a were analyzed for granzyme B expression (*n*=6 mice in each group). (d, e, and f) CD4$^+$ and CD8$^+$ T cells isolated from the lung of *PyMT-Control* and *PyMT-Lin28B* mice were interrogated for expression of IFN-γ (d), TNF-α (e), and T-bet expression (*n*=6 mice in each group). (g) CD4$^+$ T cells prepared as in panel d were analyzed for GATA-3, FoxP3, and RoRγT expression (*n*=6 mice in each group). (h-j) Infiltration of T cells (h), the activated CD44$^+$CD62L$^-$ subset (i) and the Th1 subset of the CD4$^+$ T cells (j) were analyzed in the pre-metastatic lung (5-month old) in *MMTV-Neu-Lin28B* and its control mice (*n*=6 mice in each group).

Data are presented as the mean ± SEM, and p-values were calculated using two-tailed Student's *t*-test. *$p < 0.05$, **$p < 0.01$, ns, no significance. Source data are provided as a Source Data file.

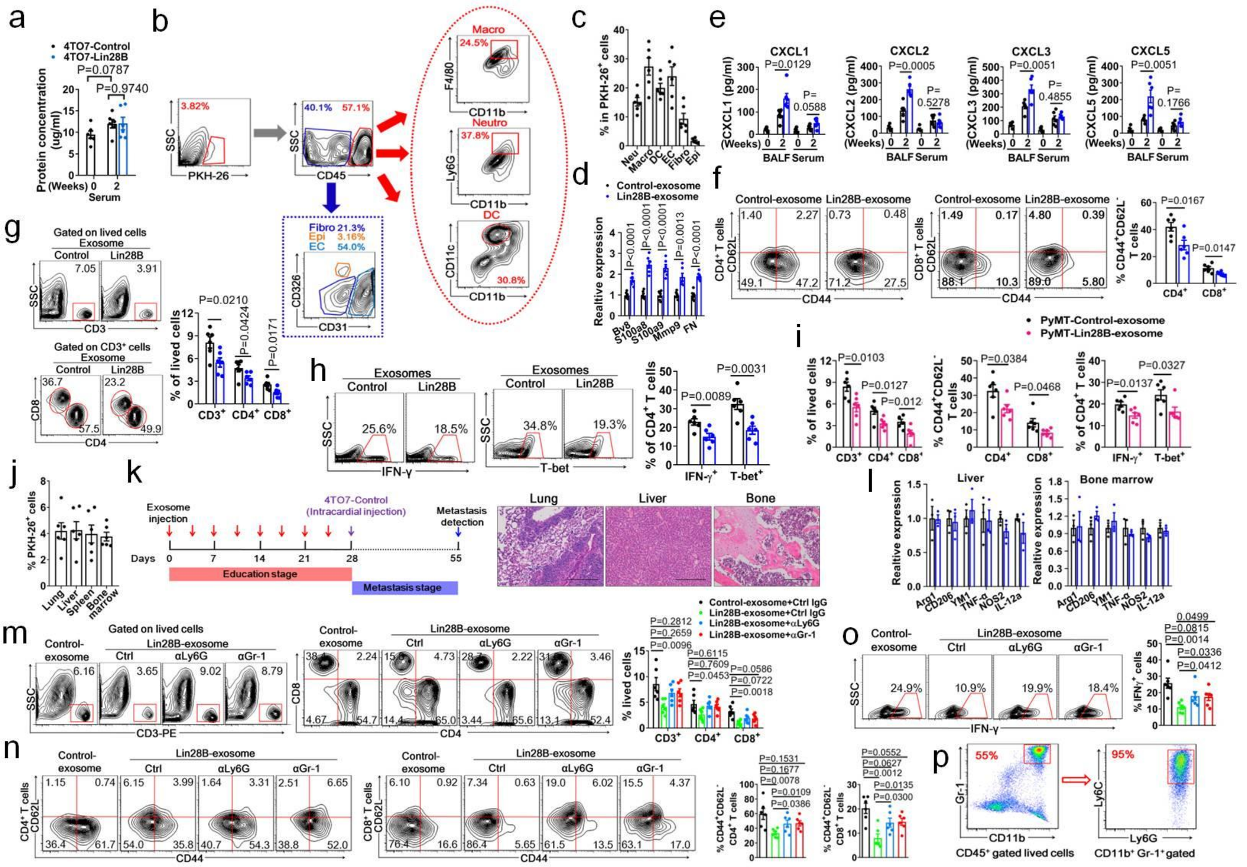

Supplementary Figure 6. Tumor-derived exosomes recapitulate the Lin28B-induced immune-suppressive pre-metastatic niche through neutrophil recruitment and N2 conversion.

(a) Exosome protein concentrations purified from sera of tumor-bearing mice (*n*=6 mice in each group). (b) Representative gating strategy used to identify PKH-26$^+$ lung cells in mice injected with PKH-26 labeled tumor-exosomes. (c) Percentage of individual cell types among PKH-26-positive cells (*n*=6 mice in each group). Neu: neutrophil; Macro: macrophage; DC: dendritic cell; EC: endothelial cell; Fibro: Fibroblast; Epi: epithelial cell. (d) Niche-characteristic gene expression was detected in the lungs of mice inoculated with the indicated exosomes (*n*=6 mice in each group). FN: fibronectin. (e) Quantification of CXCLs in BALFs and serum of mice treated with the indicated exosomes (*n*=6 mice in each group). (f-h) The indicated T cell subsets were analyzed in the lungs of mice inoculated with Lin28B-exosome or control-exosome (*n*=6 mice in each group). (i) The indicated T cell subsets were analyzed in the lungs of mice treated with PyMT-exosomes (11-week old) (*n*=6 mice in each group). (j) The experiment was the same

as in panel b, except that The PKH-26-positive cell proportion was measured in the indicated tissues (*n*=6 mice in each group). (k) Schematic illustration and H&E staining sections of intracardial injection of 4TO7-Control cells after exosome inoculation. Scale bars: 200 μm. (l) The experiment was the same as in Fig. 6c, except that the expression of N1/N2 marker genes were determined in liver and bone marrow neutrophils (*n*=3 mice in each group). (m-o) The experiment was conducted as in Fig. 6c. The indicated T cells are measured in the treated mice (*n*=6 mice in each group). (p) The $CD11b^{+}Gr\text{-}1^{+}$ subset among the $CD45^{+}$ lived cells in the pre-metastatic lung of 4TO7 tumor-bearing mice was subjected to Ly6G and Ly6C expression detection.

Data are presented as the mean ± SEM, and p-values were calculated using two-tailed Student's t-test. *p < 0.05, **p < 0.01, ***p < 0.001, ns, no significance. Source data are provided as a Source Data file.

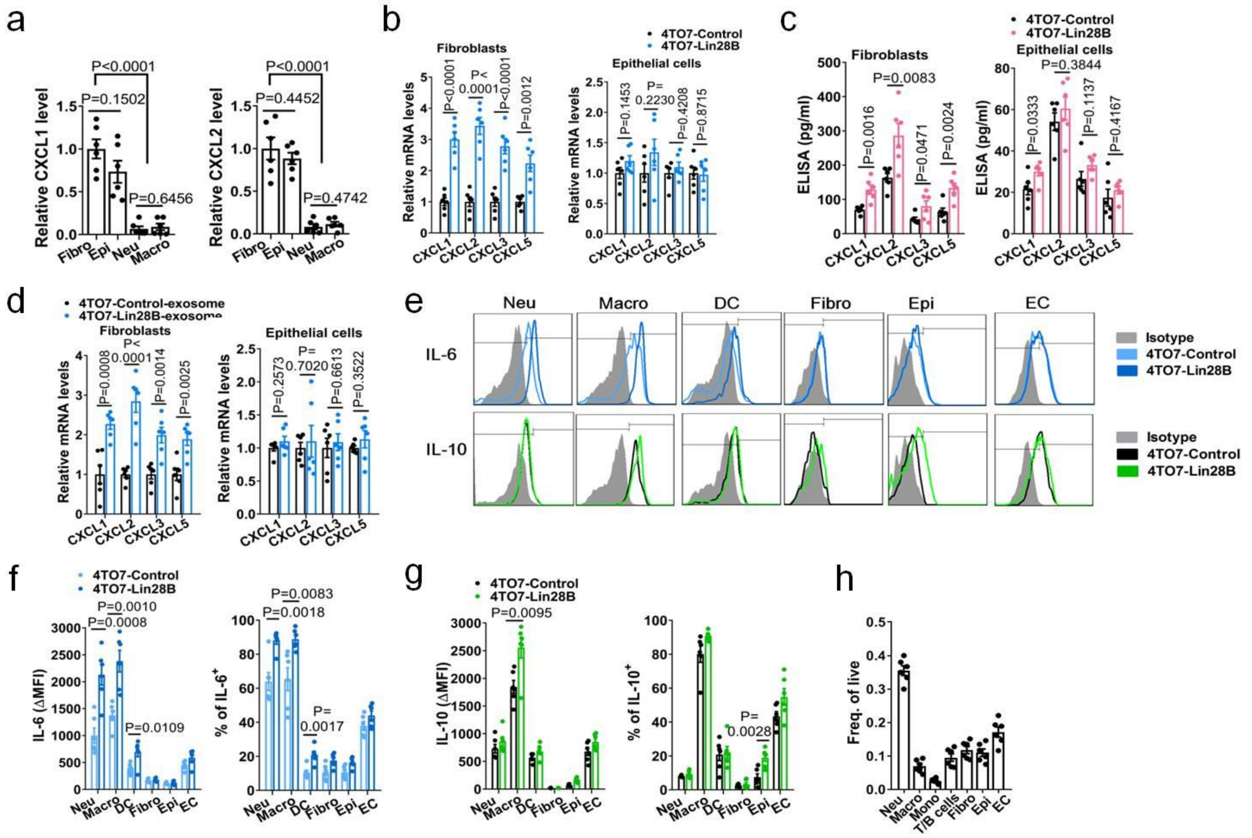

Supplementary Figure 7. Lung fibroblasts, neutrophils, and macrophages are the primary recipient cells responsible for increased CXCLs, IL-6, and IL-10 levels.

(a) The indicated cell populations were purified from the pre-metastatic lung of the 4TO7-Control mice, and *CXCL1* and *CXCL2* mRNA was quantified by qRT-PCR. GAPDH served as internal control (*n*=6 mice in each group). (b) CXCLs mRNA expression in lung fibroblasts and epithelial cells in the pre-metastatic niche. GAPDH served as internal control (*n*=6 mice in each group). (c) Fibroblasts and epithelial cells were sorted from the pre-metastatic lung of tumor-bearing mice at 3 weeks after tumor inoculation and cultured in vitro. The supernatants were collected and subjected to ELISA assay for chemokine detection (*n*=6 culturing experiments). (d) Fibroblasts and epithelial cells were sorted from the lungs of mice treated with tumor-derived exosomes, and chemokine *CXCLs* mRNA expression were detected by qRT-PCR. GAPDH served as internal control (*n*=6 mice in each group). (e) Representative flow cytometry analysis of IL-6 and IL-10 in the indicated cells in the pre-metastatic lung of tumor-bearing mice at 3 weeks after tumor inoculation. (f) Mean fluorescence intensity (MFI) of IL-6 expression and proportion of

IL-6$^+$ cells in each cell population as indicated (*n*=6 mice in each group). (g) MFI of IL-10 expression and proportion of IL-10$^+$ cells in each cell population as indicated (*n*=6 mice in each group). (h) The frequency of the main lung cell types in the pre-metastatic lung was quantified by FACS (*n*=6 mice in each group). For panel (a), (e), (f), (g) and (h), Neu: neutrophil; Macro: macrophage; DC: dendritic cell; EC: endothelial cell; Fibro: Fibroblast; Epi: epithelial cell. Data are presented as the mean ± SEM, and p-values were calculated using two-tailed Student's *t*-test. *p < 0.05, **p < 0.01, ***p < 0.001, ns, no significance. Source data are provided as a Source Data file.

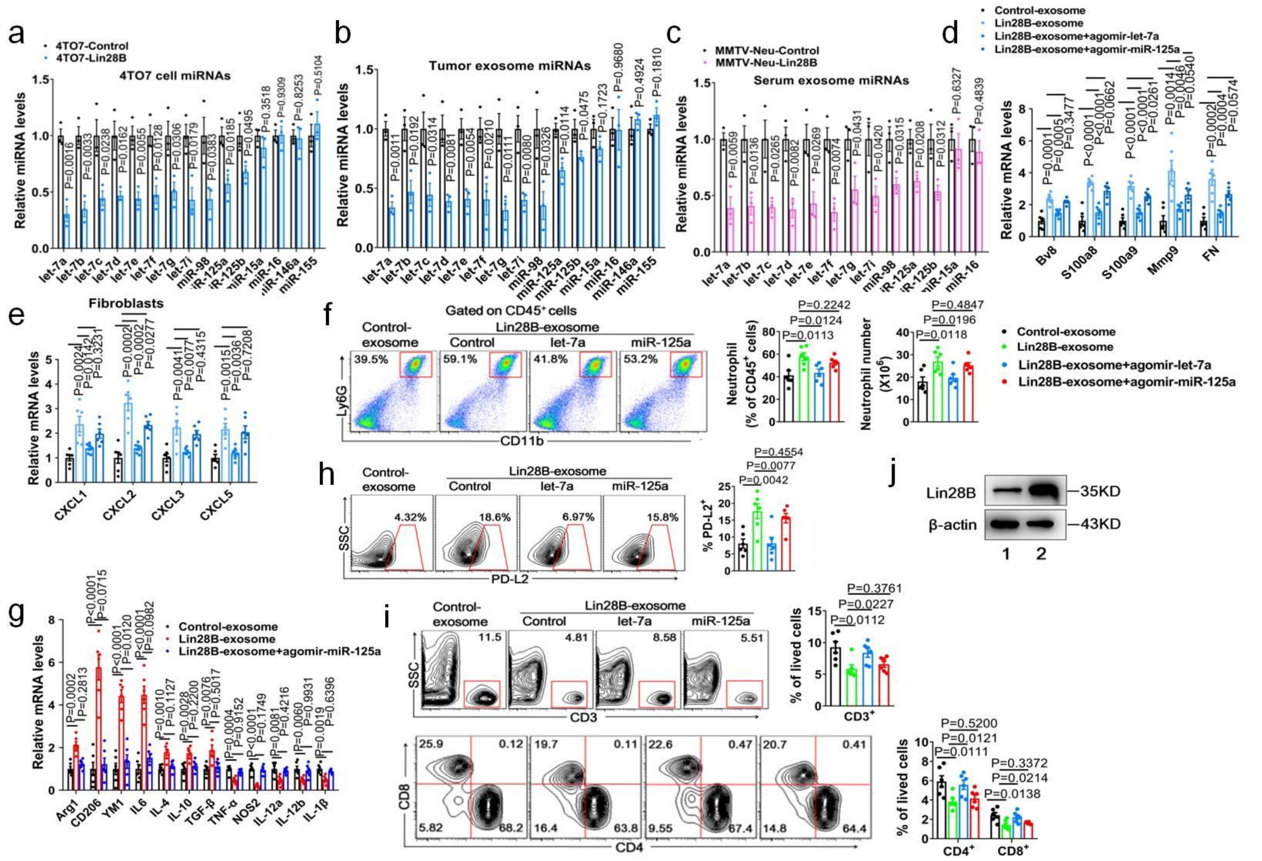

Supplementary Figure 8. A lower let-7s content in Lin28B-exosome is responsible for immune suppression.

(a) Let-7s family members were decreased in 4TO7-Lin28B cells (*n*=3 mice in each group). (b) Let-7s family members were decreased in Lin28B-expressing tumor-derived exosomes (*n*=3 mice in each group). (c) Let-7s family members were decreased in the serum exosomes in *MMTV-Neu-Lin28B* mice (*n*=3 mice in each group). (d) Let-7a inhibited increased production of niche-characteristic genes in lungs mediated by Lin28B-exosome. Exosome treatment was the same as in Fig. 6c. Na ïve mice were i.v injected with Control-exosome or Lin28B-exosome in the presence of agomir-let-7a, agomir-miR-125a or control agomir twice a week for consecutive three weeks. The total lung cells were dissociated and the lung expression of niche-characteristic genes was analyzed by qRT-PCR. GAPDH served as internal control (*n*=6 mice in each group). FN: fibronectin. (e) Let-7a inhibited increased chemokine production of fibroblasts in the pre-metastatic lung induced by Lin28B-exosomes. The experiment was the same as for panel d, except that the chemokine was detected by qRT-PCR. GAPDH served as internal control (*n*=6

mice in each group). (f) Let-7a inhibited increased neutrophil infiltration in the pre-metastatic lung induced by Lin28B-exosomes (*n*=6 mice in each group). (g) MiR-125a did not inhibit neutrophil N2 conversion in the pre-metastatic niche induced by Lin28B-exosomes (*n*=6 mice in each group). (h) Let-7a inhibited PD-L2 upregulation of lung neutrophils induced by Lin28B-exosomes (*n*=6 mice in each group). (i) Let-7a mitigated the inhibitory T cell accumulation in the pre-metastatic niche induced by Lin28B-exosomes (*n*=6 mice in each group). (j) Expression levels of Lin28B were analyzed by western blotting. β-actin served as a loading control. Results show that $ALDH^+$ 4TO7-Lin28B cells (line 2) expressed much more Lin28B than the $ALDH^-$ one (line 1). The experiment was repeated three times independently with similar results; data from one representative experiment are shown.

Data are presented as the mean ± SEM, and p-values were calculated using two-tailed Student's *t*-test. *p < 0.05, **p < 0.01, ns, no significance. Source data are provided as a Source Data file.

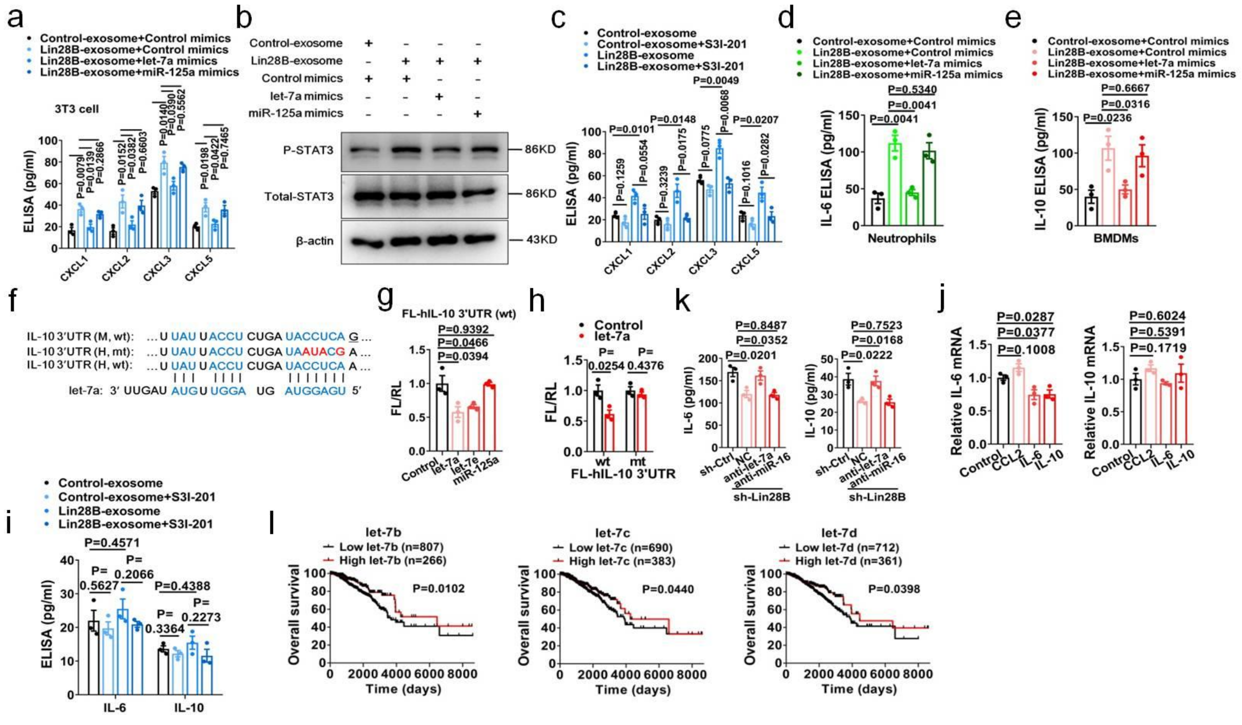

Supplementary Figure 9. A lower let-7s content in Lin28B-exosomes was determined to be responsible for increased cytokines production by in vitro cell culture assay.

(a) Let-7a rescued Lin28B-exosomes-mediated increased chemokine production in 3T3 cells (*n*=3 culturing experiments). (b) Let-7a rescued Lin28B-exosomes-mediated increased STAT3 phosphorylation in 3T3 cells. Experiment was repeated three times independently with similar results; data from one representative experiment are shown. (c) STAT3 inhibitor S3I-201 restore increased CXCLs production induced by Lin28B-exosomes in 3T3 cells (*n*=3 culturing experiments). (d-e) Let-7a rescued Lin28B-exosome-mediated increased IL-6 (d) and IL-10 (e) production (*n*=3 culturing experiments). (f) The predicted let-7a binding site was conserved in the 3′UTR of human and mouse *IL-10* mRNA. The base-paired region between let-7a and *IL-10* mRNA is signaled in blue, and the corresponding sequence in the mutated version (mt) in red. The underlined G represents the only different base between human and mouse IL-10 mRNA in this region. Aberrant: H, human; M, mouse; wt, wild-type; mt, mutated. (g) Let-7s repressed the expression of firefly luciferase (FL) reporter FL-hIL-10-wt containing human IL-10 3′UTR with wild-type (wt) let-7a binding sites (*n*=3 culturing experiments). (h) Let-7a specifically repressed luciferase activity of reporter FL-hIL-10-wt, but not FL-hIL-10-mt containing mutated let-7a

binding sites (*n*=3 culturing experiments). (i) IL-6 and IL-10 were detected in the indicated exosomes treated 3T3 cells pretreated with S3I-201 (*n*=3 culturing experiments). (j) Neutrophils were treated with the indicated factors and IL-6 and IL-10 mRNA were detected by qRT-PCR (*n*=3 culturing experiments). (k) Let-7a blocking restored the production of IL-6 and IL-10 inhibited by Lin28B knockdown (sh-Lin28B) (*n*=3 culturing experiments). NC: control inhibitor; sh-Ctrl: control shRNA. (l) Overall survival probabilities were calculated using the Kaplan-Meier method and analyzed using a log-rank test in a cohort of GDC TCGA breast cancer patients (1073 cases), with let-7b, let-7c or let-7d expression.

Data are presented as the mean ± SEM, and p-values were calculated using two-tailed Student's *t*-test. *p < 0.05, ns, no significance. Source data are provided as a Source Data file.

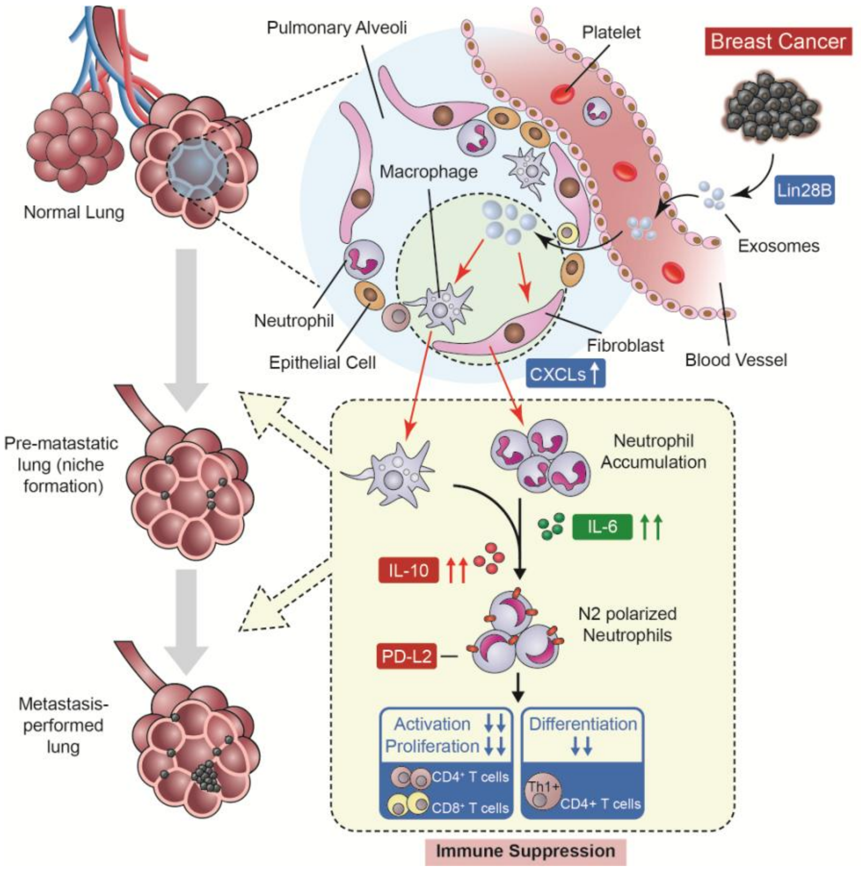

Supplementary Figure 10. The proposed model for immune-suppressive pre-metastatic niche formation in the context of Lin28B expression.

When Lin28B is expressed in breast tumors, the tumor-derived exosomes render lung fibroblasts, neutrophils and macrophages releasing increased CXCLs, IL-6 and IL-10, respectively, which are right enough in driving neutrophil recruitment and N2 conversion. With the enhanced PD-L2 and dysregulated cytokine milieus, the N2 neutrophils are then involved in $CD4^+$ and $CD8^+$ T cell inhibition, inducing immune-suppressive pre-metastatic niche and metastasis-arising.

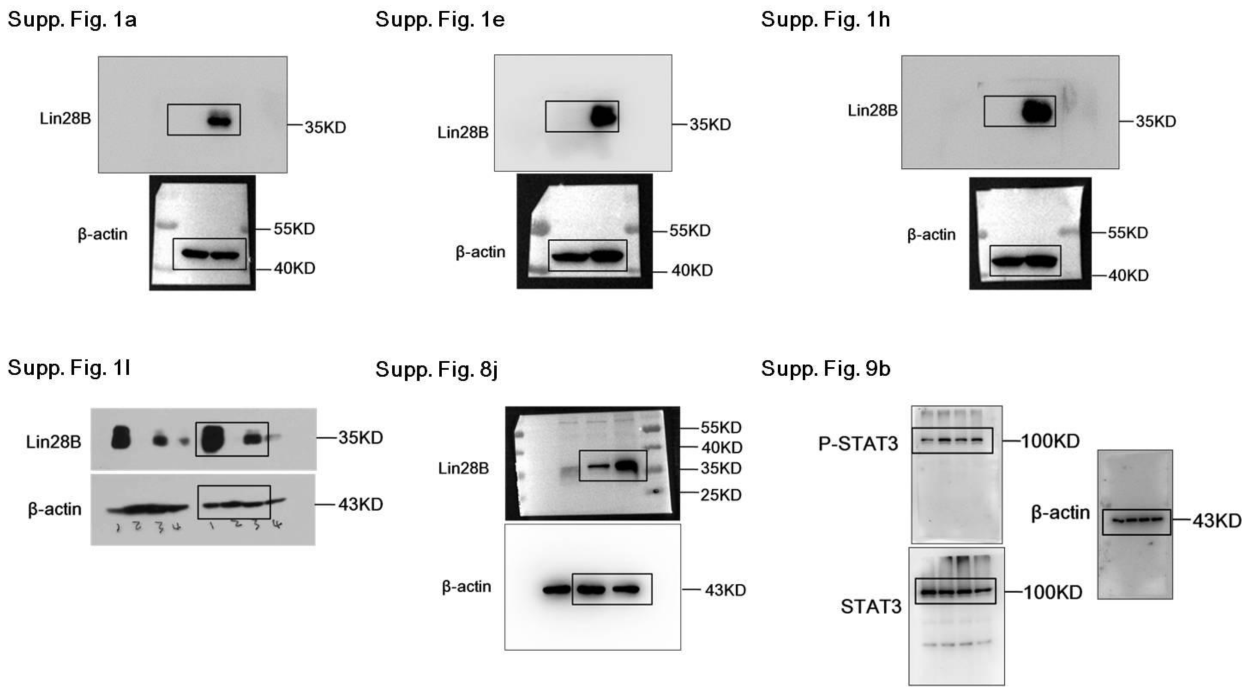

Supplementary Figure 11. Original uncropped Western Blots in Supplementary Figures.

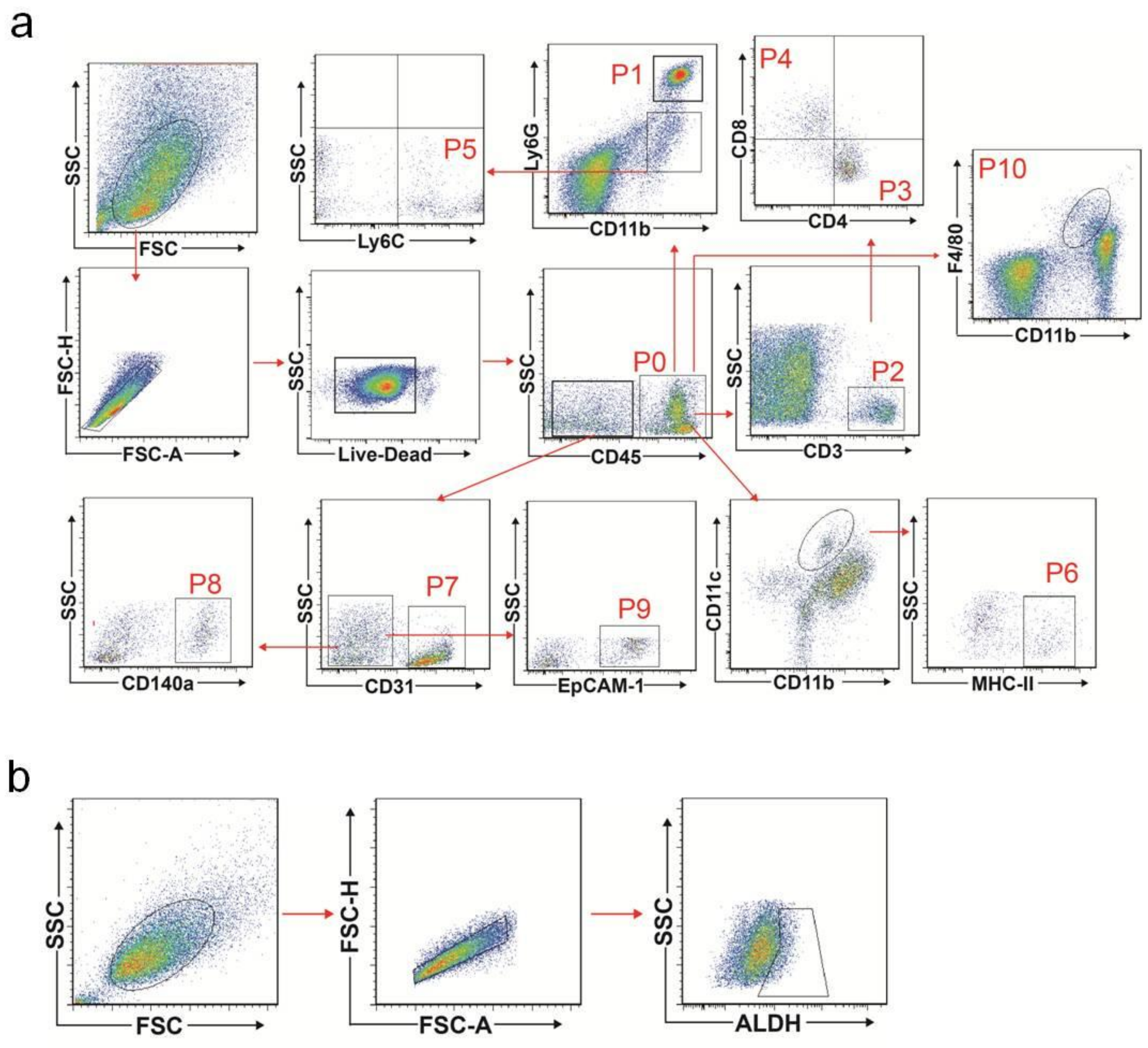

Supplementary Figure 12. Gating strategy of flow cytometric analyses.

(a) Gate strategy of CD45$^+$ (P0) was used in fig. 2b; 4a; 6k and supplementary fig. 2c, e, m; 4a-b; 5a, h; 6g, i, m, p; 8f, i. Gate strategy of CD45$^+$CD11b$^+$Ly6G$^+$ (P1) was used in fig 5a-d; 6f, i; 7c and supplementary fig. 2j-k, p; 7e-h; 8f, h. Gate strategy of CD45$^+$CD3$^+$ (P2) was used in supplementary fig. 7h. Gate strategy of CD45$^+$CD3$^+$ CD4$^+$ (P3) was used in fig. 4d; 6l; 7f and supplementary fig. 4f; 5b-c, g, i; 6f, i, n. Gate strategy of CD45$^+$CD3$^+$ CD8$^+$ (P4) was used in fig. 4d; 6l; 7f and supplementary fig. 4f; 5b-c, g, i; 6f, i, n. Gate strategy of CD45$^+$CD11b$^+$Ly6G$^-$Ly6C$^+$ (P5) was used in supplementary fig. 2g. Gate strategy of CD45$^+$CD11b^{mid}Ly6C$^+$MHC-II$^+$ (P6) was used in fig. 5c and supplementary fig. 2g; 7e-h. Gate strategy of CD45$^-$CD31$^+$ (P7) was used in supplementary fig. 7e-h. Gate strategy of

$CD45^-CD31^-CD140a^+$ (P8) was used in supplementary fig. 7e-h. Gate strategy of $CD45^-CD31^+$ $EpCAM^+$ (P9) was used in supplementary fig. 7e-h. Gate strategy of $CD45^+CD11b^+$ $F4/80^+$ (P10) was used in fig. 5c; 7d and supplementary fig. 7e-h. P1 was used in neutrophil sorting in fig. 3a-b, d, f; 4b-c; 5e-h; 6h; 7e and supplementary fig. 3a-c, e-f, h; 4d-e; 6l; 7a; 8g; 9d, j. P3 and P4 were used in $CD4^+$ and $CD8^+$ T cell sorting in fig. 4e-h; 5e-h; 6m; 7g and supplementary fig. 3a-c, e-f, h; 4g-h; 5d-f, j; 6h-i, o. P8 was used in fibroblast sorting in fig. and supplementary fig. 7a-d; 8e. P9 was used in epithelial cell sorting in supplementary fig. 7a-d. P10 was used in macrophage sorting in supplementary fig. 7a; 9e. (b) Gate strategy of $ALDH^+$ was used in supplementary fig. 1k-m.

Supplementary Table 1, Related to Fig. 1

Lin28B expression in tumor tissues of the breast cancer patient subgroups according to the clinical pathologic parameters in TMA Cohort II (140 cases).

Characteristic	All	Low Lin28	High Lin28B	Comparison Of Lin28B Levels, p value
**Clinical grade**				
II	98	72	26	
II-III	36	21	15	
III	6	2	4	**0.045***
**TNM stage**				
1	33	24	9	
2	61	44	17	
3	46	27	19	0.267
**Vascular invasion**				
No	124	86	38	
Yes	16	9	7	0.291
**Lymph node**				
No	75	55	20	
Yes	65	40	25	0·136
**Recurrent**				
No	102	71	31	
Yes	38	24	14	0.467
**Distal metastasis**				
No	128	86	42	
Yes	12	2	10	**0.001****

p value was determined by Chip-squared test.

Supplementary Table 2, Related to Fig. 1

Uni- and mult-variate Cox proportional hazard model analysis for breast cancer patients overall survival in TMA Cohort II (140 cases).

Variable	Univariate		Multivariate	
	**HR (95% CI)**	***P* value**	**HR (95% CI)**	***P* value**
**Clinical grade**		0.106		\
II-III	2.088 (0.969-4.501)	0.060	\	\
III	2.771 (0.637-12.059)	0.174	\	\
**TNM stage**		**0.005****		0.302
2	1.735 (0.470-6.409)	0.409	1.992 (0.435-9.131)	0.375
3	4.719 (1.382-16.114)	**0.013***	0.765 (0.100-5.832)	0.796
**Vascular invasion**	1.602 (0.611-4.199)	0.338	\	\
**Lymph node**	2.789 (1.270-6.128)	**0.011***	2.025 (0.455-9.010)	0.354
**Recurrence**	11.771 (5.002-27.701)	**<0.001*****	10.89 (3.747-31.658)	**<0.001*****
**Distal metastases**	6.383 (2.799-14.558)	**<0.001*****	3.165 (1.099-9.116)	**0.033***
**Lin28B expression**	3.689 (1.760-7.733)	**<0.001*****	5.802 (2.391-14.080)	**<0.001*****

p value was determined by two-sided log-rank test.

Supplementary Table 3, Related to Fig. 1

Uni- and mult-variate Cox proportional hazard model analysis for luminal breast cancer patients overall survival in TMA Cohort II and III (169 cases).

Variable	Univariate		Multivariate	
	**HR (95% CI)**	***P* value**	**HR (95% CI)**	***P* value**
**Clinical grade**		**0.006****		**0.009****
II	0.644 (0.250-1.661)	0.363	0.294(0.091-0.946)	**0.04***
III	2.879(0.901-9.204)	0.074	0.235(0.093-0.59)	**0.002****
**TNM stage**		**0.009****		0.066
2	22230 (0-3.53E75)	0.905	0 (0-5.67E211)	0.958
3	68000 (0-1.08E76)	0.894	0.306 (0.113-0.828)	**0.02***
**Lymph node**	2.699 (1.205-6.047)	**0.016***	1.415 (0.45-4.449)	0.553
**Lin28B expression**	3.076 (1.502-6.299)	**0.002****	2.484 (1.169-5.279)	**0.018***

p value was determined by two-sided log-rank test.

Supplementary Table 4, Related to Fig. 1

Uni- and mult-variate Cox proportional hazard model analysis for HER2^{+} breast cancer patients overall survival in TMA Cohort II and III (43 cases).

Variable	Univariate		Multivariate	
	**HR (95% CI)**	***P* value**	**HR (95% CI)**	***P* value**
**Clinical grade**		0.336		0.170
II-III	1.36(0.176-10.54)	0.768	1.543 (0.191-12.472)	0.684
III	3.399 (0.351-32.893)	0.291	5.076 (0.508-50.696)	0.166
**TNM stage**		0.379		\
2	2.299 (0.277-19.098)	0.441	\	\
3	3.723 (0.465-29.777)	0.215	\	\
**Lymph node**	2.161 (0.688-6.797)	0.187	3.332 (0.999-11.112)	**0.050***
**Lin28B expression**	2.983(1.059-8.403)	**0.039***	5.517 (1.775-17.142)	**0.003****

p value was determined by two-sided log-rank test.

Supplementary Table 5, Related to Fig. 1

Uni- and mult-variate Cox proportional hazard model analysis for TNBC patients overall survival in TMA Cohort II and III (60 cases).

Variable	Univariate		Multivariate	
	**HR (95% CI)**	***P* value**	**HR (95% CI)**	***P* value**
**Clinical grade**		0.890		\
II-III	1.311 (0.307-5.593)	0.714	\	\
III	1.082 (0.197-5.93)	0.928	\	\
**TNM stage**		0.127		0.486
2	2.725 (0.768-9.669)	0.121	2.285 (0.589-8.873)	0.232
3	3.666 (1.043-12.89)	**0.043***	2.145 (0.380-12.120)	0.388
**Lymph node**	1.982 (0.91-4.31)	0.084	1.606 (0.493-5.238)	0.432
**Lin28B expression**	2.589 (1.187-5.647)	**0.017***	2.667 (1.196-5.948)	**0.017***

p value was determined by two-sided log-rank test.

Supplementary Table 6, Related to Fig. 1

Metastasis sites of the clinical samples were analyzed stratificated by Lin28B expression in 204 cases from GEO dataset GSE12276.

Metastasis sites	All	Low Lin28	High Lin28B	Comparison Of Lin28B Levels, p value
**Lung metastasis**				
No	159	109	50	
Yes	45	24	21	**0.045***
	204			
**Brain metastasis**				
No	188	124	64	
Yes	16	9	7	0.300
	204			
**Bone metastasis**				
No	93	59	34	
Yes	111	74	37	0.369
	204			

p value was determined by Chip-squared test.

Supplementary Table 7, Related to Fig. 8

Metastasis sites of the clinical samples were analyzed by let-7a expression in 204 cases from GEO dataset GSE12276.

Metastasis sites	All	Low Lin28	High Lin28B	Comparison Of Lin28B Levels, p value
**Lung metastasis**				
No	159	68	91	
Yes	45	30	15	**0.005****
	204			
**Brain metastasis**				
No	188	92	96	
Yes	16	6	10	0.379
	204			
**Bone metastasis**				
No	93	51	42	
Yes	111	47	64	0.075
	204			

p value was determined by Chip-squared test.

Supplementary Table 8. Oligonucleotides used in the study		
Usage	Name	Sequence (5'-3')
For knockdown	shhLin28B-1F	CCGG GCCATTACTGTCAGAGCATCACTCGAG TGATGCTCTGACAGTAATGGCTTTTTG
	shhLin28B-1R	AATTCAAAAA GCCATTACTGTCAGAGCATCA CTCGAGTGATGCTCTGACAGTAATGGC
	shhLin28B-2F	CCGG GCCTGAGTCTGTGTGTGTACACTCGAG TGTACACACACAGACTCAGGCTTTTTG
	shhLin28B-2R	AATTCAAAAAGCCTGAGTCTGTGTGTGTACACTCG AGTGTACACACACAGACTCAGGC
	shhLin28B-3F	CCGG GCAATGCCATTACTAGCTATTCTCGAG AATAGCTAGTAATGGCATTGC TTTTTG
	shhLin28B-3R	AATTCAAAAAGCAATGCCATTACTAGCTATTCTCG AG AATAGCTAGTAATGGCATTGC
qPCR primers	mCXCL1-F	AGACTCCAGCCACACTCCAA
	mCXCL1-R	TGACAGCGCAGCTCATTG
	mCXCL2-F	AAAATCATCCAAAAGATACTGAACAA
	mCXCL2-R	CTTTGGTTCTTCCGTTGAGG
	mCXCL3-F	CCCCAGGCTTCAGATAATCA
	mCXCL3-R	TCTGATTTAGAATGCAGGTCCTT
	mCXCL5-F	TAGAGCCCCAATCTCCACAC
	mCXCL5-R	GGAGCTGGAGGCTCATTGT
	mBV8-F	TGCTACCGCTGCTGTTCA
	mBV8-R	AGTCCTTAAACACGCCAAGC
	mS100A8-F	TCCTTGCGATGGTGATAAAA
	mS100A8-R	GGCCAGAAGCTCTGCTACTC
	mS100A9-F	GACACCCTGACACCCTGAG
	mS100A9-R	TGAGGGCTTCATTTCTCTTCTC
	mMMP9-F	AGACGACATAGACGGCATCC
	mMMP9-R	TCGGCTGTGGTTCAGTTGT
	mFibronectin-F	TGTTAGCAGACCCTCCATCTTTG
	mFibronectin-R	T TCTCTTAAACAACACTGAGCAG
	mSox2-F	TCCAAAAACTAATCACAACAATCG
	mSox2-R	GAAGTGCAATTGGGATGAAAA
	mKlf-F	CGGGAAGGGAGAAGACACT
	mKlf-R	GAGTTCCTCACGCCAACG
	mMyc-F	TTTGTCTATTTGGGGACAGTGTT
	mMyc-R	CATCGTCGTGGCTGTCTG

	mNanog-F	TGCTTACAAGGGTCTGCTACTG
	mNanog-R	GAGGCAGGTCTTCAGAGGAA
	mPD-L1-F	AAATCGTGGTCCCCAAGC
	mPD-L1-R	TCCTCATGTTTTGGGAACTATCT
	mPD-L2-F	TGTGCTGCCTTTTCTGTGTC
	mPD-L2-R	GCAGCATGGTCTGTGTCAAT
	mmu-Arg1-F	GAATCTGCATGGGCAACC
	mmu-Arg1-R	GAATCCTGGTACATCTGGGAAC
	mmu-CD206-F	CCACAGCATTGAGGAGTTTG
	mmu-CD206-R	ACAGCTCATCATTTGGCTCA
	mYM1-R	AAGAACACTGAGCTAAAAACTCTCCT
	mYM1-R	GAGACCATGGCACTGAACG
	mIL-4-F	CATCGGCATTTTGAACGAG
	mIL-4-R	CGAGCTCACTCTCTGTGGTG
	mIL-6-F	TGCCTTCATTTATCCCTTGAA
	mIL-6-R	TTACTACATTCAGCCAAAAAGCAC
	mIL-10-F	CAGAGCCACATGCTCCTAGA
	mIL-10-R	TGTCCAGCTGGTCCTTTGTT
	mTGF-β-F	AAGGGCTACCATGCCAACTTCTG
	mTGF-β-R	GCACGATCATGTTGGACAGCTGC
	mTNF-α-F	TCTTCTCATTCCTGCTTGTGG
	mTNF-α-R	GAGGCCATTTGGGAACTTCT
	mNOS2-F	CTTTGCCACGGACGAGAC
	mNOS2-R	TCATTGTACTCTGAGGGCTGAC
	mIL-12a-F	GGAAGCACGGCAGCAGAATA
	mIL-12a-R	AACTTGAGGGAGAAGTAGGAATGG
	mIL-12b-F	AAGGAACAGTGGGTGTCCAG
	mIL-12b-R	GTTAGCTTCTGAGGACACATCTTG
	mIL-1β-F	AGTTGACGGACCCCAAAAG
	mIL-1β-R	AGCTGGATGCTCTCATCAGG
	mGAPDH-F	ATGTTCCAGTATGACTCCACTCAC
	mGAPDH-R	GACACCAGTAGACTCCACGACATA
	mmu-miR-146a-F	ACACTCCAGCTGGGTGAGAACTGAATTCCA
	mmu-miR-146a-R	CTCAACTGGTGTCGTGGAGTCGGCAATTCAGTTGAGAGGGATTC
	mmu-miR-155-5	ACACTCCAGCTGGGTTAATGCTAATTGTGAT

	mmu-miR-155-5p-F	
	mmu-miR-155-5p-R	CTCAACTGGTGTCGTGGAGTCGGCAATTCAGTTGAGACCCC TAT
	mmu-miR-15a-F	ACACTCCAGCTGGGTAGCAGCACATAATGG
	mmu-miR-15a-R	CTCAACTGGTGTCGTGGAGTCGGCAATTCAGTTGAGCACAAACC
	mmu-miR-16-F	ACACTCCAGCTGGGTAGCAGCACGTAAATA
	mmu-miR-16-R	CTCAACTGGTGTCGTGGAGTCGGCAATTCAGTTGAGCGCCAATA
	let-7a-F	ACACTCCAGCTGGGTGAGGTAGTAGGTTGT
	let-7a-R	CTCAACTGGTGTCGTGGAGTCGGCAATTCAGTTGAGAACTATAC
	let-7b-F	ACACTCCAGCTGGGTGAGGTAGTAGGTTGT
	let-7b-R	CTCAACTGGTGTCGTGGAGTCGGCAATTCAGTTGAGAACCACAC
	let-7c-F	ACACTCCAGCTGGGTGAGGTAGTAGGTTGT
	let-7c-R	CTCAACTGGTGTCGTGGAGTCGGCAATTCAGTTGAG AACCATAC
	let-7d-F	ACACTCCAGCTGGGAGAGGTAGTAGGTTGC
	let-7d-R	CTCAACTGGTGTCGTGGAGTCGGCAATTCAGTTGAG AACTATGC
	let-7e-F	ACACTCCAGCTGGGTGAGGTAGGAGGTTGT
	let-7e-R	CTCAACTGGTGTCGTGGAGTCGGCAATTCAGTTGAGAACTATAC
	let-7f-F	ACACTCCAGCTGGGTGAGGTAGTAGATTGT
	let-7f-R	CTCAACTGGTGTCGTGGAGTCGGCAATTCAGTTGAGAACTATAC
	let-7g-F	ACACTCCAGCTGGG TGAGGTAGTAGTTTGT
	let-7g-R	CTCAACTGGTGTCGTGGAGTCGGCAATTCAGTTGAG AACTGTAC
	let-7i-F	ACACTCCAGCTGGGTGAGGTAGTAGTTTGT
	let-7i-R	CTCAACTGGTGTCGTGGAGTCGGCAATTCAGTTGAG AACAGCAC
	miR-98-F	ACACTCCAGCTGGGTGAGGTAGTAAGTTGT
	miR-98-R	CTCAACTGGTGTCGTGGAGTCGGCAATTCAGTTGAGAACAATAC
	U6-RT	ATATGGAACGCTTCACG
	U6-F	CTCGCTTCGGCAGCACA

	Universal-R	TCAACTGGTGTCGTGGAGTCG
	mmu-miR-125a-5p-F	ACACTCCAGCTGGGTCCCTGAGACCCTTTAAC
	mmu-miR-125a-5p-R	CTCAACTGGTGTCGTGGAGTCGGCAATTCAGTTGAGTCACAGGT
	mmu-miR-125b-5p-F	ACACTCCAGCTGGGTCCCTGAGACCCTAAC
	mmu-miR-125b-5p-R	CTCAACTGGTGTCGTGGAGTCGGCAATTCAGTTGAGTCACAAGT
For cloning primers	IL-10-3'UTR-m1-F	ATATTTATTACCTCTGATACCTCGACCCCCATTTCTATTTATTTAC
	IL-10-3'UTR-m1-R	GTAAATAAATAGAAATGGGGGTCGAGGTATCAGAGGTAATAAATAT
	IL-10-3'UTR-m2-F	ATATTTATTACCTCTGATACCACGACCCCCATTTCTATTTATTTAC
	IL-10-3'UTR-m2-R	GTAAATAAATAGAAATGGGGGTCGTGGTATCAGAGGTAATAAATAT
	IL-10-3'UTR-m3-F	ATATTTATTACCTCTGATACTACGACCCCCATTTCTATTTATTTAC
	IL-10-3'UTR-m3-R	GTAAATAAATAGAAATGGGGGTCGTAGTATCAGAGGTAATAAATAT
	IL-10-3'UTR-m4-F	ATATTTATTACCTCTGATAATACGACCCCCATTTCTATTTATTTAC
	IL-10-3'UTR-m4-R	GTAAATAAATAGAAATGGGGGTCGTATTATCAGAGGTAATAAATAT
	FL into PcDNA3.0-UP	ATGCGGCCGCATGGAAGACGCCAAAAACATAAAGAAAGG
	FL into PcDNA3.0-Down	CGGAGCTCTTACACGGCGATCTTTCCGCCCTTC
	hIL-10-3'UTR-PcDNA3.0-UP	CGCTCGAGGACATCAGGGTGGCGACTCTATAG
	hIL-10-3'UTR-PcDNA3.0-Down	GCAGATCTCTGCTATGAAGACAGACAAACAATG
	hLin28B-pBABE-UP	AC GGATCC ATGGCCG AAGGCGGGGC TAGCAAAG
	hLin28B-pBABE-Down	GC GTCGAC TTATGTCTTTTTCCTTTTTTGAACTGAAGGCC
miRNA	agomir-let-7a-5p	UGAGGUAGUAGGUUGUAUAGUU

mimics used in vivo assay	agomir-125a-5p	UCCCUGAGACCCUUUAACCUGUGA
miRNA mimics used in cell experiments	mmu-let-7a-mimics	UGAGGUAGUAGGUUGUAUAGUU
	mmu-let-7c-mimics	UGAGGUAGUAGGUUGUAUGGUU
	mmu-let-7e-mimics	UGAGGUAGGAGGUUGUAUAGUU
	anti-mmu-let-7a	AACUAUACAACCUACUACCUCA
	anti-mmu-miR-125a	UCACAGGUUAAAGGGUCUCAGGA
