# Supplementary material for: Lin28B-high breast cancer cells promote immune suppression in the lung pre-metastatic niche via exosomes and support cancer progression
Source: Nat Commun. 2022 Feb 16;13:897. doi: 10.1038/s41467-022-28438-x (PMC8850492; doi:10.1038/s41467-022-28438-x)
Supplement: Supplementary file 2 — Reporting Summary [file 41467_2022_28438_MOESM2_ESM.pdf]

nature portfolio

Corresponding author(s): Lixing Zhan

Last updated by author(s): Dec 26, 2021

Reporting Summary

Nature Portfolio wishes to improve the reproducibility of the work that we publish. This form provides structure for consistency and transparency in reporting. For further information on Nature Portfolio policies, see our Editorial Policies and the Editorial Policy Checklist.

Statistics

For all statistical analyses, confirm that the following items are present in the figure legend, table legend, main text, or Methods section.

n/a	Confirmed	
[ ]	[x]	The exact sample size (*n*) for each experimental group/condition, given as a discrete number and unit of measurement
[ ]	[x]	A statement on whether measurements were taken from distinct samples or whether the same sample was measured repeatedly
[ ]	[x]	The statistical test(s) used AND whether they are one- or two-sided<br>*Only common tests should be described solely by name; describe more complex techniques in the Methods section.*
[x]	[ ]	A description of all covariates tested
[ ]	[x]	A description of any assumptions or corrections, such as tests of normality and adjustment for multiple comparisons
[ ]	[x]	A full description of the statistical parameters including central tendency (e.g. means) or other basic estimates (e.g. regression coefficient) AND variation (e.g. standard deviation) or associated estimates of uncertainty (e.g. confidence intervals)
[ ]	[x]	For null hypothesis testing, the test statistic (e.g. *F*, *t*, *r*) with confidence intervals, effect sizes, degrees of freedom and *P* value noted<br>*Give P values as exact values whenever suitable.*
[x]	[ ]	For Bayesian analysis, information on the choice of priors and Markov chain Monte Carlo settings
[x]	[ ]	For hierarchical and complex designs, identification of the appropriate level for tests and full reporting of outcomes
[ ]	[x]	Estimates of effect sizes (e.g. Cohen's *d*, Pearson's *r*), indicating how they were calculated

Our web collection on statistics for biologists contains articles on many of the points above.

Software and code

Policy information about availability of computer code

Data collection	BD FACSAriaTM II flow cytometer was used to run samples and data was acquired and analyzed by BD FACSDiva software. In vivo bioluminescence signal was assessed and analyzed with the IVIS Spectrum In Vivo Imaging System (Perkin Elmer). Immunofluorescence images were acquired using confocal laser microscopy (Carl Zeiss).
Data analysis	Flow cytometry data were analyzed with BD FACSDiva software. Bioluminescence was analyzed using the IVIS Imaging System (Perkin Elmer). Image J was used for image quantification analysis. GraphPad Prism ver 6.0 and SPSS Statistics ver19 were used for statistical analysis.

For manuscripts utilizing custom algorithms or software that are central to the research but not yet described in published literature, software must be made available to editors and reviewers. We strongly encourage code deposition in a community repository (e.g. GitHub). See the Nature Portfolio guidelines for submitting code & software for further information.

Data

Policy information about availability of data

All manuscripts must include a data availability statement. This statement should provide the following information, where applicable:

- Accession codes, unique identifiers, or web links for publicly available datasets
- A description of any restrictions on data availability
- For clinical datasets or third party data, please ensure that the statement adheres to our policy

The small RNA sequencing data of 4TO7 tumor exosomes, Related to Fig. 6D, were deposited in GEO database (GSE185588) in Page 17 in manuscript. All the data were released, and everyone can download it for free.

Field-specific reporting

Please select the one below that is the best fit for your research. If you are not sure, read the appropriate sections before making your selection.

- [x] Life sciences
- [ ] Behavioural & social sciences
- [ ] Ecological, evolutionary & environmental sciences

For a reference copy of the document with all sections, see nature.com/documents/nr-reporting-summary-flat.pdf

Life sciences study design

All studies must disclose on these points even when the disclosure is negative.

Sample size	No sample size calculation was done either for in vivo or in vitro studies. For in vivo studies, n=5-20 mice per group is sufficient to detect meaningful biological differences with good reproducibility. For in vitro studies, all the experiments were replicated at least for 3 individual, independent experiments.
Data exclusions	No data were excluded in our studies.
Replication	All experiments were performed at least three independent times and/or with sufficient cells/animals per group to demonstrate statistical significance.
Randomization	For cell experiments, all cells in each experiment were from the same pool of parental cells. All mice were age- and sex-matched (female mice). For in vivo experiments, animals were randomized based on tumour burden before they were assigned into different treatment groups. All animals were maintained in the same environment and handled by the same procedure.
Blinding	For data collected by objective instruments, such as plate readers, qPCR cyclers, microscopy software, flow cytometers, animal IVIS systems, and western blotting, the investigators were not blinded to group allocation during data collection. Laboratory personnel was blinded to animal randomization to drug treatment, which was performed by the PI. However, the laboratory personnel was blinded during the data analysis from each individual mice. Data analyses were performed by the Biostatistician who were blinded to experimental groups.

Reporting for specific materials, systems and methods

We require information from authors about some types of materials, experimental systems and methods used in many studies. Here, indicate whether each material, system or method listed is relevant to your study. If you are not sure if a list item applies to your research, read the appropriate section before selecting a response.

Materials & experimental systems

n/a	Involved in the study	
☐	☒	Antibodies
☐	☒	Eukaryotic cell lines
☒	☐	Palaeontology and archaeology
☐	☒	Animals and other organisms
☐	☒	Human research participants
☒	☐	Clinical data
☒	☐	Dual use research of concern

Methods

n/a	Involved in the study	
☒	☐	ChIP-seq
☐	☒	Flow cytometry
☒	☐	MRI-based neuroimaging

Antibodies

Antibodies used

APC-Cyanine7 CD45 monoclonal antibody (I3/2.3) (Thermo Fisher Scientific, A15395),
FITC CD11b monoclonal antibody (M1/70) (Thermo Fisher Scientific, 11-0112-82),
eFluor 450 Ly6G monoclonal antibody (1A8-Ly6g) (Thermo Fisher Scientific, 48-9668-82), eFluor 450 F4/80 monoclonal antibody (BM8) (Thermo Fisher Scientific, 48-4801-82),
PE CD3e monoclonal antibody (145-2C11) (Thermo Fisher Scientific, 12-0031-82),
FITC CD4 monoclonal antibody (GK1.5) (Thermo Fisher Scientific, 11-0041-82),
APC CD8a monoclonal antibody (53-6.7) (Thermo Fisher Scientific, 17-0081-82),
eFluor 450 CD44 monoclonal antibody (IM7) (Thermo Fisher Scientific, 48-0441-82), PerCP-Cyanine5.5 CD62L (L-Selectin) monoclonal antibody (MEL-14) (Thermo Fisher Scientific, 45-0621-82),
PerCP-eFluor 710 CD273 (B7-DC) monoclonal antibody (122) (Thermo Fisher Scientific, 46-9972-82),
PerCP-eFluor 710, CD274 (PD-L1, B7-H1) monoclonal antibody (MIH1) (Thermo Fisher Scientific, 46-5983-42),
FITC CD31 (PECAM-1) monoclonal antibody (390) (Thermo Fisher Scientific, 11-0311-81),
APC CD140a (PDGFRA) monoclonal antibody (APA5) (Thermo Fisher Scientific, 17-1401-81), APC CD326 (EpCAM) monoclonal antibody (G8.8) (Thermo Fisher Scientific, 17-5791-82),
PE IL-6 monoclonal antibody (MP5-20F3) (Thermo Fisher Scientific, 12-7061-81),
PE IL-10 monoclonal antibody (JES5-16E3) (Thermo Fisher Scientific, 12-7101-81),

PE IFN gamma monoclonal antibody (XMG1.2) (Thermo Fisher Scientific, 12-7311-81),
PE T-bet monoclonal antibody (4B10) (Thermo Fisher Scientific, 12-5825-80),
PE GATA-3 monoclonal antibody (TWAJ) (Thermo Fisher Scientific, 12-9966-41),
PE FoxP3 monoclonal antibody (FJK16s) (Thermo Fisher Scientific, 12-5773-80),
PE ROR gamma (t) monoclonal antibody (AFKJS-9) (Thermo Fisher Scientific, 12-6988-80),
PE Granzyme B monoclonal antibody (NGZB) (Thermo Fisher Scientific, 12-8898-80),
PE CD80 (B7-1) monoclonal antibody (16-10A1) (Thermo Fisher Scientific, 12-0801-81),
PE CD86 (B7-2) monoclonal antibody (GL1) (Thermo Fisher Scientific, 12-0862-81),
PE CD252 (OX40 Ligand) monoclonal antibody (RM134L) (Thermo Fisher Scientific, 12-5905-81),
PE CD275 (B7-H2) monoclonal antibody (HK5.3) (Thermo Fisher Scientific, 12-5985-81), CD16/CD32 monoclonal antibody (93) (Thermo Fisher Scientific Cat# 14-0161-82),
APC Ly6C monoclonal antibody (HK1.4) (Thermo Fisher Scientific, 17-5932-82),

Phospho-Stat3 (Tyr705) (D3A7) XP® Rabbit mAb (Cell Signaling technology, 9145S),
Stat3 (79D7) Rabbit mAb (Cell Signaling technology, 4904S),
β-actin (13E5) Rabbit mAb (Cell Signaling technology, 4970S),
Alix (3A9) Mouse mAb (Cell Signaling technology, 2171S),
Annexin V Antibody (Cell Signaling technology, 8555S),
HSP70 (D69) Antibody (Cell Signaling technology, 4876S),
Flotillin-1 (D2V7J) XP® Rabbit mAb (Cell Signaling technology, 18634S), anti-mouse IgG, HRP-linked antibody (Cell Signaling technology, 7076S), anti-rabbit IgG, HRP-linked antibody (Cell Signaling technology, 7074S),
Rabbit Polyclonal To Human LIN28B (LifeSpan BioScience, LS-B3423),
BD PharmingenTM Purified rat anti-mouse Ly6G (1A8) (BD Bioscience, 551459),
rabbit anti-mouse CD45 mAb (D3F8Q) (Cell Signaling technology, 70257S),
rabbit anti-mouse CD11b mAb (E6E1M) (Cell Signaling technology, 17800S),
rat anti-mouse Ly6C monoclonal antibody (ER-MP20) (Abcam, ab54223),
rabbit anti-mouse F4/80 mAb (CI:A3-1) (Bio-Rad, MCA497),
Alexa Fluor 488 donkey anti-rabbit IgG (Invitrogen, A-21206),
Alexa Fluor 594 donkey anti-rat IgG (Invitrogen, A-21209),
Alexa Fluor 488 donkey anti-rat IgG (Invitrogen, A-21208),
InVivo mAb anti-mouse CD3e (Bio X Cell, Clone 145-2C11, BE-0001-1),
InVivo mAb anti-mouse CD28 (Bio X Cell, Clone 37.51, BE-0015-1),
In vivoPlus anti-mouse IL-4 (Bio X Cell, Clone 11B11, BE-0015-1)
InVivo mAb anti-mouse IL-6 (Bio X cells, Clone MP5-20F3, BE0046)
InVivo mAb anti-mouse IL-10 (Bio X cells, Clone JES5-2A5, BE0049)
InVivo mAb anti-mouse Ly6G (Bio X cells, Clone 1A8, BE0075-1)
InVivoPlus rat IgG2a isotype control (Bio X cells, Clone 2A3, BP0089)
In vivoPlus anti-mouse Ly6G/Ly6C (Gr-1) (Bio X cells, Clone RB6-8C5, BP0075)
InVivo mAb anti-mouse PD-L2 (B7-DC) (Bio X cells, Clone TY25, BE0112)
InVivo mAb anti-mouse IL-12 p40 (Bio X Cell, Clone C17.8, BE0051)

Validation

All antibodies were validated according to respective manufacturer's information. Anti-Lin28B Ab (LifeSpan BioScience, LS-B3423) was validated with Lin28B-expressing transgenic model (Supplementary Fig. S1a, e and h) and Lin28B knockdown MDA-MB-231 cells (Supplementary Fig. S1l).
.APC-Cyanine7 CD45 monoclonal antibody (I3/2.3) (Thermo Fisher Scientific, A15395), mouse, FC, https://www.thermofisher.cn/cn/zh/antibody/product/CD45-Antibody-clone-I3-2-3-Monoclonal/A15395
FITC CD11b monoclonal antibody (M1/70) (Thermo Fisher Scientific, 11-0112-82), mouse, IHC, IHC (F), FC, Neu, https://www.thermofisher.cn/cn/zh/antibody/product/CD11b-Antibody-clone-M1-70-Monoclonal/11-0112-82
eFluor 450 Ly6G monoclonal antibody (1A8-Ly6g) (Thermo Fisher Scientific, 48-9668-82), mouse, IHC ICC/IF, FC, https://www.thermofisher.cn/cn/zh/antibody/product/Ly-6G-Antibody-clone-1A8-Ly6g-Monoclonal/48-9668-82
eFluor 450 F4/80 monoclonal antibody (BM8) (Thermo Fisher Scientific, 48-4801-82), mouse, IHC, IHC (P), IHC (F), IHC (PFA), ICC/IF, FC, https://www.thermofisher.cn/cn/zh/antibody/product/F4-80-Antibody-clone-BM8-Monoclonal/48-4801-82
PE CD3e monoclonal antibody (145-2C11) (Thermo Fisher Scientific, 12-0031-82), mouse, IHC (F), FC, https://www.thermofisher.cn/cn/zh/antibody/product/CD3e-Antibody-clone-145-2C11-Monoclonal/12-0031-82
FITC CD4 monoclonal antibody (GK1.5) (Thermo Fisher Scientific, 11-0041-82), mouse, IHC, IHC (P), IHC (F), ICC/IF, FC, https://www.thermofisher.cn/cn/zh/antibody/product/CD4-Antibody-clone-GK1-5-Monoclonal/11-0041-82
APC CD8a monoclonal antibody (53-6.7) (Thermo Fisher Scientific, 17-0081-82), mouse, IHC, IHC (P), ICC/IF, FC, https://www.thermofisher.cn/cn/zh/antibody/product/CD8a-Antibody-clone-53-6-7-Monoclonal/17-0081-82
eFluor 450 CD44 monoclonal antibody (IM7) (Thermo Fisher Scientific, 48-0441-82), human, mouse, IHC, ICC/IF, FC, https://www.thermofisher.cn/cn/zh/antibody/product/CD44-Antibody-clone-IM7-Monoclonal/48-0441-82
PerCP-Cyanine5.5 CD62L (L-Selectin) monoclonal antibody (MEL-14) (Thermo Fisher Scientific, 45-0621-82), mouse, rat, FC, https://www.thermofisher.cn/cn/zh/antibody/product/CD62L-L-Selectin-Antibody-clone-MEL-14-Monoclonal/45-0621-82
PerCP-eFluor 710 CD273 (B7-DC) monoclonal antibody (122) (Thermo Fisher Scientific, 46-9972-82), mouse, FC, https://www.thermofisher.cn/cn/zh/antibody/product/CD273-B7-DC-Antibody-clone-122-Monoclonal/46-9972-82
PerCP-eFluor 710, CD274 (PD-L1, B7-H1) monoclonal antibody (MIH1) (Thermo Fisher Scientific, 46-5983-42), human, IHC (F), FC, https://www.thermofisher.cn/cn/zh/antibody/product/CD274-PD-L1-B7-H1-Antibody-clone-MIH1-Monoclonal/46-5983-42
FITC CD31 (PECAM-1) monoclonal antibody (390) (Thermo Fisher Scientific, 11-0311-81), human, mouse, fish, WB, IHC, IHC (P), IHC

(F), ICC/IF, FC, https://www.thermofisher.cn/cn/zh/antibody/product/CD31-PECAM-1-Antibody-clone-390-Monoclonal/11-0311-81
APC CD140a (PDGFRA) monoclonal antibody (APA5) (Thermo Fisher Scientific, 17-1401-81), human, mouse, IHC (F), ICC/IF, FC, https://www.thermofisher.cn/cn/zh/antibody/product/CD140a-PDGFRA-Antibody-clone-APA5-Monoclonal/17-1401-81
APC CD326 (EpCAM) monoclonal antibody (G8.8) (Thermo Fisher Scientific, 17-5791-82), human, mouse, IHC, IHC (F), ICC/IF, FC, https://www.thermofisher.cn/cn/zh/antibody/product/CD326-EpCAM-Antibody-clone-G8-8-Monoclonal/17-5791-82
PE IL-6 monoclonal antibody (MP5-20F3) (Thermo Fisher Scientific, 12-7061-81), mouse, FC, https://www.thermofisher.cn/cn/zh/antibody/product/IL-6-Antibody-clone-MP5-20F3-Monoclonal/12-7061-81
PE IL-10 monoclonal antibody (JES5-16E3) (Thermo Fisher Scientific, 12-7101-81), human, mouse, FC, https://www.thermofisher.cn/cn/zh/antibody/product/IL-10-Antibody-clone-JES5-16E3-Monoclonal/12-7101-81
PE IFN gamma monoclonal antibody (XMG1.2) (Thermo Fisher Scientific, 12-7311-81), human, mouse, IHC, ICC/IF, FC, Neu, https://www.thermofisher.cn/cn/zh/antibody/product/IFN-gamma-Antibody-clone-XMG1-2-Monoclonal/12-7311-81
PE T-bet monoclonal antibody (4B10) (Thermo Fisher Scientific, 12-5825-80), human, mouse, rhesus monkey, FC, https://www.thermofisher.cn/cn/zh/antibody/product/T-bet-Antibody-clone-eBio4B10-4B10-Monoclonal/12-5825-80
PE GATA-3 monoclonal antibody (TWAJ) (Thermo Fisher Scientific, 12-9966-41), human, mouse, pig, rhesus monkey, FC, https://www.thermofisher.cn/cn/zh/antibody/product/Gata-3-Antibody-clone-TWAJ-Monoclonal/12-9966-41
PE FoxP3 monoclonal antibody (FJK16s) (Thermo Fisher Scientific, 12-5773-80), mouse, rat, cat, dog, bovine, pig, WB, IHC, IHC (F), ICC/IF, FC, https://www.thermofisher.cn/cn/zh/antibody/product/FOXP3-Antibody-clone-FJK-16s-Monoclonal/12-5773-80
PE ROR gamma (t) monoclonal antibody (AFKJS-9) (Thermo Fisher Scientific, 12-6988-80), human, mouse, pig, rhesus monkey, IHC (F), FC, https://www.thermofisher.cn/cn/zh/antibody/product/ROR-gamma-t-Antibody-clone-AFKJS-9-Monoclonal/12-6988-80
PE Granzyme B monoclonal antibody (NGZB) (Thermo Fisher Scientific, 12-8898-80), mouse, FC, https://www.thermofisher.cn/cn/zh/antibody/product/Granzyme-B-Antibody-clone-NGZB-Monoclonal/12-8898-80
PE CD80 (B7-1) monoclonal antibody (16-10A1) (Thermo Fisher Scientific, 12-0801-81), dog, mouse, pig, IHC (P), ICC/IF, FC, https://www.thermofisher.cn/cn/zh/antibody/product/CD80-B7-1-Antibody-clone-16-10A1-Monoclonal/12-0801-81
PE CD86 (B7-2) monoclonal antibody (GL1) (Thermo Fisher Scientific, 12-0862-81), human, mouse, FC, ELISA, https://www.thermofisher.cn/cn/zh/antibody/product/CD86-B7-2-Antibody-clone-GL1-Monoclonal/12-0862-81
PE CD252 (OX40 Ligand) monoclonal antibody (RM134L) (Thermo Fisher Scientific, 12-5905-81), human, mouse, FC, https://www.thermofisher.cn/cn/zh/antibody/product/CD252-OX40-Ligand-Antibody-clone-RM134L-Monoclonal/12-5905-81
PE CD275 (B7-H2) monoclonal antibody (HK5.3) (Thermo Fisher Scientific, 12-5985-81), mouse, FC, Neu, https://www.thermofisher.cn/cn/zh/antibody/product/CD275-B7-H2-Antibody-clone-HK5-3-Monoclonal/12-5985-82
CD16/CD32 monoclonal antibody (93) (Thermo Fisher Scientific Cat# 14-0161-82), Hamster, Human, Mouse, Rat, IHC, IHC (P), IHC (F), FC, BLOCK, https://www.thermofisher.cn/cn/zh/antibody/product/CD16-CD32-Antibody-clone-93-Monoclonal/14-0161-82
APC Ly6C monoclonal antibody (HK1.4) (Thermo Fisher Scientific, 17-5932-82), mouse, FC, https://www.thermofisher.cn/cn/zh/antibody/product/Ly-6C-Antibody-clone-HK1-4-Monoclonal/17-5932-82
Phospho-Stat3 (Tyr705) (D3A7) XP® Rabbit mAb (Cell Signaling technology, 9145S), human, mouse, rat, monkey, WB, IP, IHC, IF, F, CHIP, https://www.cellsignal.cn/products/primary-antibodies/phospho-stat3-tyr705-d3a7-xp-rabbit-mab/9145?site-search-type=Products&N=4294956287&Ntt=9145&fromPage=plp&_requestid=2359830
Stat3 (79D7) Rabbit mAb (Cell Signaling technology, 4904S), human, mouse, rat, monkey, WB, IP, CHIP, https://www.cellsignal.cn/products/primary-antibodies/stat3-79d7-rabbit-mab/4904?site-search-type=Products&N=4294956287&Ntt=4904&fromPage=plp&_requestid=2343070
β-actin (13E5) Rabbit mAb (Cell Signaling technology, 4970S), human, mouse, rat, monkey, pig, WB, IHC, IF, F, https://www.cellsignal.cn/products/primary-antibodies/b-actin-13e5-rabbit-mab/4970?site-search-type=Products&N=4294956287&Ntt=4970&fromPage=plp&_requestid=2348819

Alix (3A9) Mouse mAb (Cell Signaling technology, 2171S), human, mouse, rat, monkey, WB, IP, https://www.cellsignal.cn/products/primary-antibodies/alix-3a9-mouse-mab/2171?site-search-type=Products&N=4294956287&Ntt=2171&fromPage=plp&_requestid=2342885
Annexin V Antibody (Cell Signaling technology, 8555S), human, mouse, rat, monkey, WB, https://www.cellsignal.cn/products/primary-antibodies/annexin-v-antibody/8555?site-search-type=Products&N=4294956287&Ntt=8555&fromPage=plp&_requestid=2349099
HSP70 (D69) Antibody (Cell Signaling technology, 4876S), human, mouse, rat, monkey, WB, https://www.cellsignal.cn/products/primary-antibodies/hsp70-d69-antibody/4876?site-search-type=Products&N=4294956287&Ntt=4876&fromPage=plp&_requestid=2349145
Flotillin-1 (D2V7J) XP® Rabbit mAb (Cell Signaling technology, 18634S), human, mouse, rat, WB, IP, IHC, IF, https://www.cellsignal.cn/products/primary-antibodies/flotillin-1-d2v7j-xp-rabbit-mab/18634?site-search-type=Products&N=4294956287&Ntt=18634s&fromPage=plp&_requestid=2342510
anti-mouse IgG, HRP-linked antibody (Cell Signaling technology, 7076S), WB, https://www.cellsignal.cn/products/secondary-antibodies/anti-mouse-igg-hrp-linked-antibody/7076?site-search-type=Products&N=4294956287&Ntt=7076&fromPage=plp&_requestid=2342603
anti-rabbit IgG, HRP-linked antibody (Cell Signaling technology, 7074S), WB, https://www.cellsignal.cn/products/secondary-antibodies/anti-rabbit-igg-hrp-linked-antibody/7074?site-search-type=Products&N=4294956287&Ntt=7074&fromPage=plp&_requestid=2342680
Rabbit Polyclonal To Human LIN28B (LifeSpan BioScience, LS-B3423), human, IHC, IHC (P), IF, WB, FC, https://www.lsbio.com/antibodies/ihc-plus-lin28b-antibody-aa1-30-flow-if-immunofluorescence-ihc-wb-western-ls-b3423/106095
BD PharmingenTM Purified rat anti-mouse Ly6G (1A8) (BD Bioscience, 551459), rat, IHC (P), IHC (Fr), FC, IP, https://www.bdbiosciences.com/en-us/search-results?searchKey=551459
rabbit anti-mouse CD45 mAb (D3F8Q) (Cell Signaling technology, 70257S), mouse, IP, IHC, IF, https://www.cellsignal.cn/products/primary-antibodies/cd45-d3f8q-rabbit-mab/70257?site-search-type=Products&N=4294956287&Ntt=70257s&fromPage=plp&_requestid=2342127
rabbit anti-mouse CD11b mAb (E6E1M) (Cell Signaling technology, 17800S), mouse, WB, IP, IF, https://www.cellsignal.cn/products/primary-antibodies/cd11b-itgam-e6e1m-rabbit-mab/17800?site-search-type=Products&N=4294956287&Ntt=17800s&fromPage=plp&_requestid=2342250
rat anti-mouse Ly6C monoclonal antibody (ER-MP20) (Abcam, ab54223), mouse, IHC-Fr, FC, https://www.abcam.com/ly6c-antibody-

er-mp20-ab54223.html
rabbit anti-mouse F4/80 mAb (CI:A3-1) (Bio-Rad, MCA497), mouse, IP, IF, WB, https://www.bio-rad-antibodies.com/monoclonal/mouse-f4-80-antibody-cl-a3-1-mca497.html?f=Purified&_ga=2.165775674.504521805.1638962755-426116405.1638962753
Alexa Fluor 488 donkey anti-rabbit IgG (Invitrogen, A-21206), Rabbit, IHC, IHC (F), IHC (P), IHC(Free), ICC/IF, FC, WB, https://www.thermofisher.cn/cn/zh/antibody/product/Donkey-anti-Rabbit-IgG-H-L-Highly-Cross-Adsorbed-Secondary-Antibody-Polyclonal/A-21206
Alexa Fluor 594 donkey anti-rat IgG (Invitrogen, A-21209), Rat, IHC, IHC (P), IHC (Free), ICC/IF, FC, https://www.thermofisher.cn/cn/zh/antibody/product/Donkey-anti-Rat-IgG-H-L-Highly-Cross-Adsorbed-Secondary-Antibody-Polyclonal/A-21209
Alexa Fluor 488 donkey anti-rat IgG (Invitrogen, A-21208), Rat, IHC, IHC(F), ICC/IF, FC, https://www.thermofisher.cn/cn/zh/antibody/product/Donkey-anti-Rat-IgG-H-L-Highly-Cross-Adsorbed-Secondary-Antibody-Polyclonal/A-21208
InVivo mAb anti-mouse CD3e (Bio X Cell, Clone 145-2C11, BE-0001-1), mouse, in vitro T cell stimulation/activation, IF, FC, WB, in vivo T cell depletion, https://bxcell.com/product/m-cd3e
InVivo mAb anti-mouse CD28 (Bio X Cell, Clone 37.51, BE-0015-1), mouse, in vitro T cell stimulation/activation, in vivo CD28 blockade, https://bxcell.com/product/m-cd28
In vivoPlus anti-mouse IL-4 (Bio X Cell, Clone 11B11, BE-0015-1), mouse, in vivo IL-4 neutralization, in vitro IL-4 neutralization, in vivo IL-4 receptor stimulation, FC, Western blot, in vivo T cell depletion, https://bxcell.com/product/invivoplus-anti-m-il-4
InVivo mAb anti-mouse IL-6 (Bio X cells, Clone MP5-20F3, BE0046), mouse, in vivo IL-6 neutralization, in vitro IL-6 neutralization, https://bxcell.com/product/m-il-6
InVivo mAb anti-mouse IL-10 (Bio X cells, Clone JES5-2A5, BE0049), mouse, in vivo IL-10 neutralization, in vitro IL-10 neutralization, https://bxcell.com/product/m-il-10
InVivo mAb anti-mouse Ly6G (Bio X cells, Clone 1A8, BE0075-1), mouse, in vivo neutrophil depletion, in vivo MDSC depletion, IF, IHC-P, IHC-Fr, FC, https://bxcell.com/product/invivomab-anti-m-ly-6g
InVivoPlus rat IgG2a isotype control (Bio X cells, Clone 2A3, BP0089), https://bxcell.com/product/invivoplus-rat-igg2a-isotype-control-anti-trinitrophenol
In vivoPlus anti-mouse Ly6G/Ly6C (Gr-1) (Bio X cells, Clone RB6-8C5, BP0075), mouse, in vivo depletion of Gr-1+ myeloid cells, FC, IHC-P (paraffin), IHC-Fr
InVivo mAb anti-mouse PD-L2 (B7-DC) (Bio X cells, Clone TY25, BE0112), mouse, in vivo PD-L2 blockade, in vitro PD-L2 blockade, IHC-Fr, FC, https://bxcell.com/product/m-b7-dc-pd-l2
InVivo mAb anti-mouse IL-12 p40 (Bio X Cell, Clone C17.8, BE0051), mouse, in vivo IL-12p40 neutralization, p40 affinity chromatography, IP, ELISA, FC, WB, https://bxcell.com/product/invivomab-anti-m-il-12-il-23

Eukaryotic cell lines

Policy information about cell lines

Cell line source(s)	4TO7, 3T3, 293T, MDA-MB-231 and 293-GPG cells are all from our lab preserved.
Authentication	Cells were authenticated through Short Tandem Repeat (STR) analysis.
Mycoplasma contamination	All cell lines in our laboratory are routinely tested for mycoplasma contamination and cells used in this study are negative for mycoplasma.
Commonly misidentified lines (See ICLAC register)	No commonly misidentified cell lines were used in the study.

Animals and other organisms

Policy information about studies involving animals; ARRIVE guidelines recommended for reporting animal research

Laboratory animals	BALB/c and C57BL/6 mice (female, 4-6 wks old) were purchased from Shanghai SLAC Laboratory Animal Corporation (Shanghai, China) and used in the animal experiments. Lin28B knock-in mice (Lin28BKI) in background of C57BL/6 were generated by Cyagen Biosciences Inc. through CRISPR/Cas9-mediated genome editing (Fig. S1D). MMTV-Cre mice in background of C57BL/6 were purchased from Shanghai Biomodel Organism Co., Ltd. MMTV-PyMT mice in background of FVB/n were purchased from Nanjing GemPharmatech Co., Ltd. MMTV-PyMT mice were backcrossed into the C57BL/6 strain to N8, and then intercrossed with MMTV-Cre and Lin28BKI mice, to obtain PyMT; MMTV-Cre (PyMT-Control) and PyMT; MMTV-Cre; Lin28BKI (PyMT-Lin28B) mice. MMTV-Neu transgenic mice, which express an activated rat c-Neu oncogene (Erbb2) in a FVB/n background, were provided by G.-H. Hu (Shanghai Institute of Nutrition and Health, University of Chinese Academy of Sciences, Chinese Academy of Sciences, Shanghai, China). MMTV-Neu mice were backcrossed into the C57BL/6 strain to N8, and then intercrossed with MMTV-Cre and Lin28BKI mice, to obtain MMTV-Neu; MMTV-Cre (MMTV-Neu) and MMTV-Neu; MMTV-Cre; Lin28BKI mice (MMTV-Neu-Lin28B). The OT-1 mice were purchased from The Jackson Laboratory (Cat #003831).
Wild animals	This study did not involve wild animals.
Field-collected samples	This study did not involve samples collected from field.
Ethics oversight	All animal studies were conducted according to the guidelines for the care and use of laboratory animals and were approved by Institutional Biomedical Research Ethics Committee of Shanghai Institute of Nutrition and Health.

Note that full information on the approval of the study protocol must also be provided in the manuscript.

Human research participants

Policy information about studies involving human research participants

Population characteristics	The breast cancer tissues used in tumor microarray chips (female patients, age at 29-87 years) for Lin28B IHC, let-7a in situ hybridization, their correlation analysis and Kaplan–Meier survival analysis were obtained from the Biobank Center of National Engineering Center for Biochip at Shanghai (also known as Shanghai Outdo Biotech Company, Ltd). The fresh tumor tissues, para-tumor specimens, serum samples and prognostic information from female patients (age at 28-82 years) diagnosed with breast cancer and treated in Tongji Hospital of Huazhong University of Science and Technology were used in tumor and exosome miRNA detection and western blotting analysis.
Recruitment	Samples and prognostic information were obtained with informed patient consent. Samples were randomly selected, there is no potential self-selection bias or other biases. Patients treated in the year of 2019-2021 in Tongji Hospital of Huazhong University of Science and Technology with available freshly frozen tumor or serum exosome samples and sufficient prognostic information were included.
Ethics oversight	Samples and prognostic information were obtained with informed patient consent and the approval from Research Review Boards of Insititute of Nutrition and Health and Tongji Hospital of Huazhong University of Science and Technology.

Note that full information on the approval of the study protocol must also be provided in the manuscript.

Flow Cytometry

Plots

Confirm that:

- [ ] The axis labels state the marker and fluorochrome used (e.g. CD4-FITC).
- [ ] The axis scales are clearly visible. Include numbers along axes only for bottom left plot of group (a 'group' is an analysis of identical markers).
- [x] All plots are contour plots with outliers or pseudocolor plots.
- [ ] A numerical value for number of cells or percentage (with statistics) is provided.

Methodology

Sample preparation	The pre-metastatic lung tissues at 2 or 3 weeks after tumor inoculation were collected, were cut into small pieces, and incubated with dissociation solution containing 2 mg/ml collagenase type IV (Sigma), 1 mg/ml dispase (Roche) and 2 U/ml DNase I (STEMCELL Technologies) at 37℃ for 30 min. The solution was pipetting every 5 min during the incubation to make the digestion homogenous. Then, the suspension was passed through a 70-µm filter and treated with Ammonium chloride red cell-lysis buffer. For surface staining, cells were stained with antibodies conjugated with fluorochromes after Fcγ block. For intracellular staining, cells were incubated in culture medium containing (PMA) (50 ng/ml; Sigma) and ionomycin (500 ng/ml; Sigma) for 2 h, and then added to Brefeldin A (10 ug/ml; Sigma) for another 4h at 37°C. Subsequent surface staining and intracellular staining were performed using Cytofix/Cytoperm kit (BD PharMingen).
Instrument	BD FACS AriaTM II (with three lasers)
Software	BD FACSDiva software
Cell population abundance	When cells were sorted or enriched, the purity was confirmed by flow cytometry and in each case the purity was above 90%.
Gating strategy	The cells were gated on FSC-A/SSC-A basis on the location known to contain lymphocytes and myeloid cells. Doublets were excluded based on FSC-A/FSC-H gating. Lived CD45+ cells were gated for analysis of either T cells or myeloid cells. Endogenous CD4+ and CD8+ T cells were gated on CD45+CD3+CD4+ or CD45+CD3+CD8+ and analyzed for phenotype and cytokine production. Neutrophils were gated on CD45+CD11b+Ly6G+ and analyzed for phenotype. Macrophages, monocytes and dendritic cells were gated on CD45+CD11b+F4/80+, CD45+Ly6G− Ly6C+ and CD45+CDllc+MHCII+, respectively.

- [ ] Tick this box to confirm that a figure exemplifying the gating strategy is provided in the Supplementary Information.
