# Supplementary material for: Lin28B-high breast cancer cells promote immune suppression in the lung pre-metastatic niche via exosomes and support cancer progression
Source: Nat Commun. 2022 Feb 16;13:897. doi: 10.1038/s41467-022-28438-x (PMC8850492; doi:10.1038/s41467-022-28438-x)
Supplement: Supplementary file 4 — Description of Additional Supplementary Files [file 41467_2022_28438_MOESM4_ESM.pdf]

**Title:** Supplementary Data 1

**Description:** Describing the clinical information of the patients for their samples used in tumor tissue microarray assay (Fig. 1a-b, d-h, 8g-h; Supplementary Table 1-5) and western blotting (Fig. 1c). This file also contains the metastatic information of 204 patients obtained from GSE dataset GSE12276 (Supplementary Table 6-7), and our tumor exosome sequencing data related to Figure. 6d.
